# Computing metasurfaces for all-optical image processing: a brief review

**DOI:** 10.1515/nanoph-2021-0823

**Published:** 2022-02-24

**Authors:** Shanshan He, Ruisi Wang, Hailu Luo

**Affiliations:** Laboratory for Spin Photonics, School of Physics and Electronics, Hunan University, Changsha 410082, China

**Keywords:** all-optical image processing, computing metasurface, edge detection, microscopy imaging, optical differential operation, quantum imaging

## Abstract

Computing metasurfaces are two-dimensional artificial nanostructures capable of performing mathematical operations on the input electromagnetic field, including its amplitude, phase, polarization, and frequency distributions. Rapid progress in the development of computing metasurfaces provide exceptional abilities for all-optical image processing, including the edge-enhanced imaging, which opens a broad range of novel and superior applications for real-time pattern recognition. In this paper, we review recent progress in the emerging field of computing metasurfaces for all-optical image processing, focusing on innovative and promising applications in optical analog operations, image processing, microscopy imaging, and quantum imaging.

## 1 Introduction

Image processing is to perform certain specific analysis and processing of image information to meet the needs of human practical applications and visual psychology. It is an important auxiliary tool in communication technology. There are two main types of technologies for acquiring and processing optical images: digital image processing and optical image processing. The outstanding feature of an optical image is visible to the naked eye. It is an image that records the brightness, color, and position of the target object by changing physical quantities such as light intensity and wavelength. Since the changes of various physical quantities are continuous, optical images are also called analog images. Optical image processing is a technology that uses optical means and devices to complete analog computing processing and transmission of image information. To objectively measure the imaging quality of the system by characterizing the optical transfer function, spectrum analysis and filtering is an important research field [1, 2]. The foundation of this technology is based on Fourier optics [3], which realizes expected image processing functions such as edge extraction, feature recognition, and encryption [4], [5], [6], [7], [8]. Optical image processing is characterized by its real-time processing capability, up to the speed of light. At the same time, optical analog computing has natural parallel operation characteristics [9], [10], [11].

A digital image actually uses a set of digital matrices to objectively depict objects. Any one of the smallest digital units in the matrix constitutes the pixels of the image. It discretizes the continuous analog image into a regular grid, and uses a computer to digitally record the brightness information of each grid point on the image. The essence of a digital image is a matrix that stores numbers, which is a series of data. Thence, digital image processing is also called computer image processing, but generally it must be combined with software algorithms and hardware co-processing. It has the advantages of high processing accuracy, flexibility, easy adjustment of parts, and complex nonlinear processing [12]. However, this technology has the disadvantages of high hardware requirements and relatively slow speed [13], [14], [15]. For real-time processing, stringent hardware conditions are usually required, and it is difficult to meet actual use in terms of cost and time. Digital processing lacks intuition and is difficult to understand for humans, which makes it inseparable from analog technology. In practice, it is often necessary to combine the recognition characteristics of human and computer for photoelectric combined processing. In addition, the speed and power consumption of standard electronic components are difficult to greatly improve, because the feature size of integrated electronic circuits is close to the quantum limit. Therefore, research on new photonic devices provides a potential way to overcome these limitations [16].

The rapid development of nanotechnology has promoted the processing and manufacturing, scientific research, and industrial applications of micro–nano structures. The optical properties of micro–nano structures have been one of the research hotspots in the field of frontier optics, which has driven emerging disciplines such as nanophotonics, surface plasmon optics, metasurface, metamaterial optics, topological photonics, and non-Hermitian optics. The micro–nano preparation process has laid an important technical foundation for realizing high-precision and omni-directional control of light. In recent years, the development of optical metasurfaces has allowed light to be precisely regulated in the sub-wavelength thickness range [17]. It can replace bulky and bulky traditional optical components to achieve various optical functions, opening a new path for the development of compact, lightweight, and multi-functional flat-panel optical systems [18], [19], [20], [21]. By changing the lateral size, shape and arrangement of the micro–nano structure, the amplitude, phase, and polarization of the light can be effectively manipulated [22], [23], [24], [25], [26], [27], [28], [29], [30].

In this review, we introduced the working principle and implementation approach of the all-optical edge detection method based on computing metasurface. The advantages and disadvantages of computing metasurface to realize edge detection are discussed, which can provide references for the design of all-optical edge detection systems in the future. Optical metamaterials are composed of periodically arranged sub-wavelength resonance units, and their two-dimensional (2D) form becomes an optical metasurface. We call a metasurface that can perform mathematical operations on the phase, amplitude, frequency, and polarization state of the incident light field as a computing metasurface. The differential operations of the incident light field correspond to the edge detection of the optical image, so the optical analog operation is the core of the all-optical image processing technology. Compared with traditional bulky optical analog differentiators, metasurface-based spatial differentiators have the advantage of being simpler and more compact. Integration of focusing and differentiation capabilities onto a monolithic metasurface through asymmetric photonic spin–orbit interactions, further reducing the complexity of edge detection systems [31]. Edge detection is the first step in target detection, feature classification, and data compression [32], [33], [34], [35]. It can extract and retain important feature information of objects and significantly reduce the amount of data to be processed. Therefore, edge detection or enhancement has excellent application prospects for computer vision [36], [37], [38], microscope imaging [39], [40], [41], and quantum communication [42, 43].

## 2 Computing metasurfaces

Traditional optical equipment accumulates these changes based on reflection, refraction, or diffraction effects in the medium to manipulate light [44], [45], [46]. Therefore, the volume of traditional equipment is limited by the propagation effect. In recent years, the limitation of light propagation has been broken by introducing characteristic changes in the operating wavelength range [22, 47, 48]. Metamaterials are artificial materials in nanoscale designed to produce optical properties that are hardly imagined in nature. Optical metasurfaces comprise a class of optical metamaterials with a reduced dimensionality that demonstrate exceptional abilities for controlling the flow of light beyond that offered by conventional, planar interfaces between two natural materials [47].

**Figure 1:**
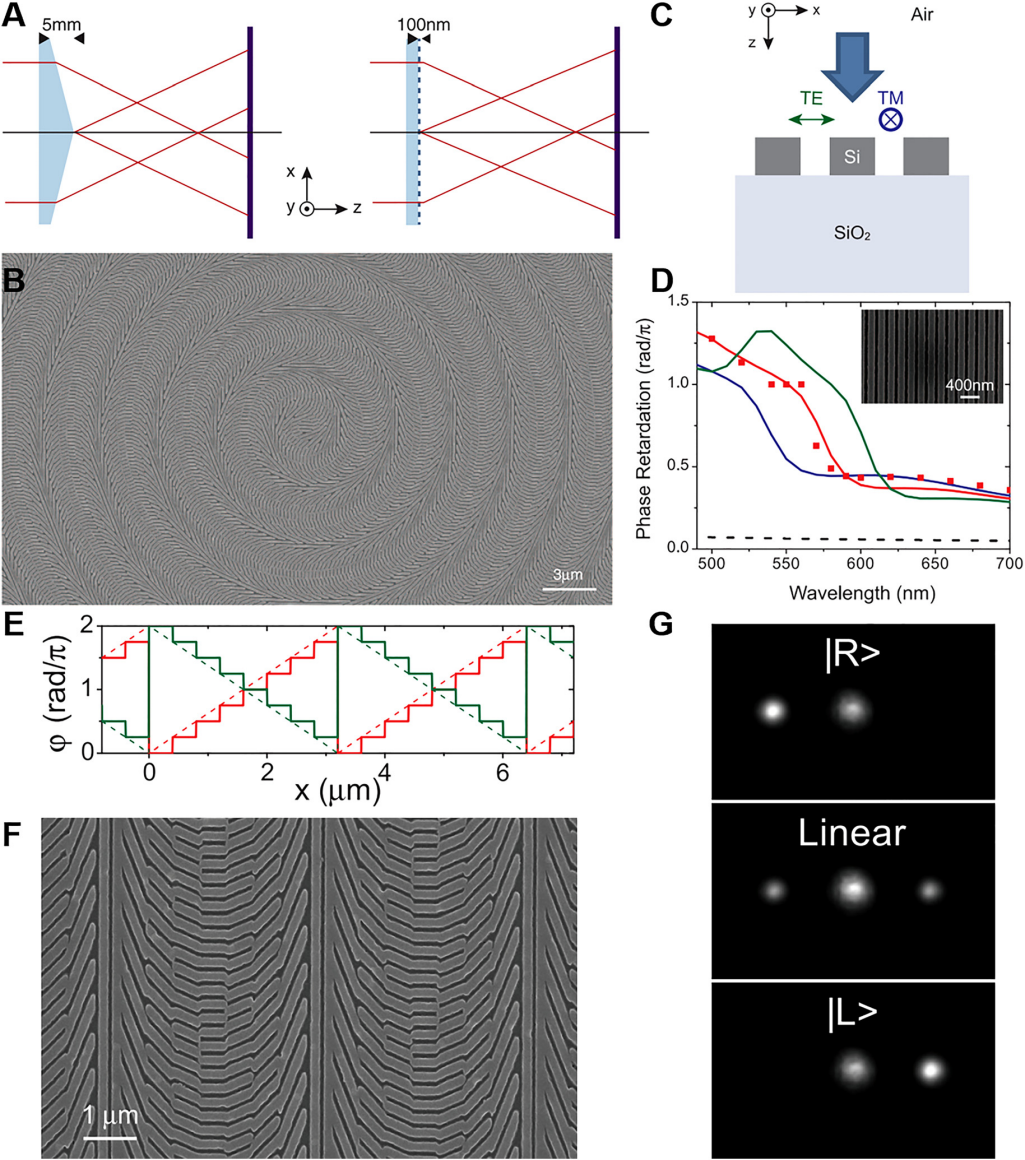
Three dielectric gradient metasurface optical elements (DGMOE) for the manipulation of light [49]. (A) Schematic of a conventional glass axicon generating a Bessel beam (left) and a DGMOE axicon featuring an ultrathin patterned layer of silicon on a quartz substrate (right). (B) Scanning electron microscopy (SEM) image of the fabricated DGMOE axicon constructed from Si nanoantennas. (C) Schematic view of a periodic 120-nm-wide and 100-nm-high Si nanobeam array on fabricated ultrathin wave plates. (D) Simulated phase retardation of a TM-polarized wave with respect to a TE-polarized wave for nanobeam arrays with widths of 100 nm (blue), 120 nm (red), 140 nm (green), with the same thickness of 100 nm and duty cycle of 60% . Measurements of the phase retardation (red squares) obtained with an array of 120-nm widths (SEM image in inset) show good agreement with the simulations. The phase retardation of the wave plate varies from 0.4π to 1.2π with the wavelength from 490 to 700 nm. In contrast, the phase retardation is approximately equal to 0.05π for a 100-nm-thick calcite film (dashed black line). (E) Discretized (solid line) and continuous (dashed line) phase profile of a DGMOE grating for illumination with LCP light (red) and RCP light (green). (F) SEM image of the fabricated grating. (G) Measured diffraction patterns from the grating under illumination with right circular polarization (top), linear polarization (middle), and left circular polarization (bottom) at λ=550 nm.

Metasurfaces can be divided into two types: the scattering type based on metallic nanomaterials and the transmissive type based on nanostructures of dielectric materials. The high diffraction efficiency of the metasurface composed of nano-metal optical antennas is limited to operation in reflection mode. The medium-gradient metasurface optical element can achieve high efficiency in the transmission mode of the visible spectrum. By patterning a 100-nm-thick silicon layer into a dense arrangement of silicon nanobeam antennas, ultrathin gratings, wave plates, and axicons have been realized [49], which is shown in Figure 1. Through the geometric structure of its structural unit, the metasurface exhibits unprecedented degrees of freedom in the polarization and phase manipulation of light, especially on the wavelength scale [50], [51], [52], [53], [54], [55], [56]. This has been applied in optics such as vortex beam generators [50, 54], metalenses [57, 58], and optical holography [59, 60]. These novel optical elements also provide considerable potential for manipulating the angular moment of light and the photonic spin Hall effect, thereby providing convenient opportunities for spin photonics and nanophotonics [61], [62], [63], [64].

By leveraging metamaterials and compressive imaging, a low-profile aperture capable of microwave imaging without lenses, moving parts, or phase shifters is demonstrated. This designer aperture allows image compression to be performed on the physical hardware layer rather than in the postprocessing stage, thus averting the detector, storage, and transmission costs associated with full diffraction-limited sampling of a scene [65]. Further, computational imaging and metasurface optics principles are combined to construct single metalens system that generates in-focus images under white light illumination [66]. This solves the problem that conventional imaging components continuously mitigate aberrations due to their bulky shortcomings, and the diffractive nature of the device can cause severe chromatic aberration.

**Figure 2:**
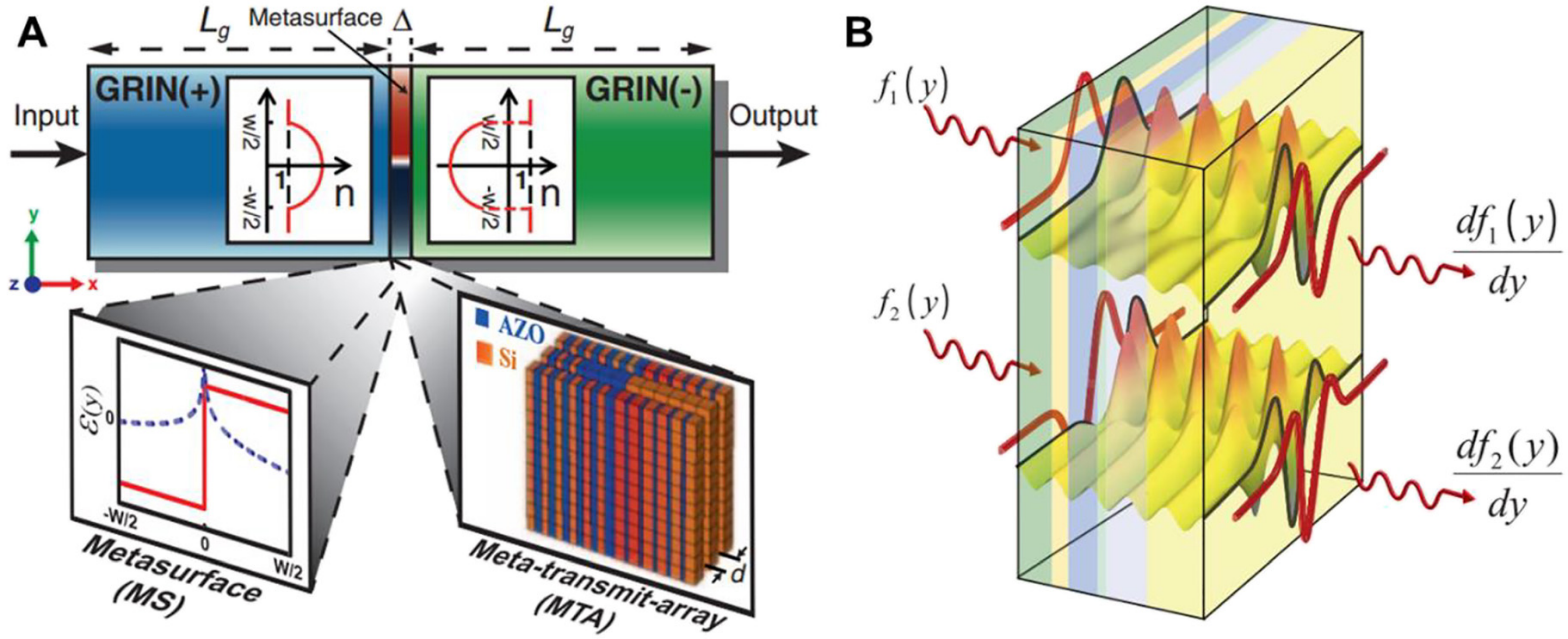
Wave-based computational metamaterials [18]. (A) The computational metamaterial system consists of three cascaded sub-blocks: (i) the Fourier transform sub-block GRIN(+), (ii) a suitably customized metasurface (MS) spatial filter in the Fourier domain, and (iii) Fourier inverse transform sub-block GRIN(−). The length $L_{\text{g}}$ of GRIN(+) and GRIN(−) is $12\lambda_{0}$ . (+) and (−), respectively, indicate that the permittivity and permeability are positive and negative. GRIN(−) has the inverse function of GRIN(+). The thin metastructure screen with thickness $\Delta =\lambda_{0}/3$ and width $W\approx 10\lambda_{0}$ is sandwiched between two GRIN structures with positive and negative parameters. Two designs are proposed for the middle metastructure: a thin MS formed of a single layer with prescribed dielectric constant and permeability [left inset, real part (red) and imaginary part (blue) with material parameters] and a realistic metatransmit-array (MTA) is formed by three sub-layers made of two alternating materials (Si and AZO). Their volume filling fractions have a properly regulated non-uniform distribution, and they have the required loss to provide the desired attenuation (right inset). (B) The metamaterial block performs a conceptual illumination of the required mathematical operations in the spatial Fourier domain. Input arbitrary wave signals propagate through the thin planar metamaterial block, in which the first-order differential operation can be performed, and a waveform satisfying the operation can be generated at output.

Computing metasurfaces refer to two-dimensional artificial nanostructures, which capable of perform mathematical operations on the input electromagnetic field, including its amplitude, phase, polarization, and frequency distributions. The spatial optical analog computing modulates the input light field in the spatial frequency domain to realize specific spatial mathematical operations. Different mechanisms involved in computing metasurfaces and pave new pathways for all-optical image processing. In 2014, Silva et al. first proposed and realized analog optical computing by designing the permittivity and permeability of the metamaterials (in Figure 2). When the incident wave passes through the metamaterial, mathematical operations are performed by directly manipulating the propagating light wave by changing the electromagnetic response of each layer of the metamaterial. It enables the expected mathematical operation between the cross-sections of the output wave and the input wave (such as spatial differentiation, integration, or convolution) [18]. Therefore, the computing metasurface offers the possibility of a miniaturized, potentially integrable wave-based computing system that is orders of magnitude thinner than traditional lens-based optical signal and data processors.

The electromagnetic waves are excited by electrical and magnetic resonance, resulting in dramatic phase changes. Relying on the spatial dispersion of the electric dipole resonance generated by the silicon nanodisk in the metasurface, the displacement of the electric dipole resonance with increasing incidence angle provides an available mechanism for spatial differentiation and edge detection [67]. In the near-infrared spectral region, amorphous silicon rods are arranged in a hexagonal manner on a silicon dioxide substrate to realize a metasurface of electromagnetic Mie-type resonance. Due to the resonant nature of the metasurface and its angle-dependent transmittance, high wave vectors can be completely transmitted while low wave vectors are filtered out. Image edge detection was achieved in magnetic resonance mode (polarization-independent) at 1400 nm and electric dipole mode at 1570 nm [68].

Based on waveguide resonance, the first or second order differential operations of the transmitted field are realized by using simple all-dielectric metal oxides to fabricate periodic ultrathin metasurfaces, or fabricating high-contrast subwavelength gratings on quartz substrates [69, 70]. It is found that the transfer function can excite the waveguide mode around a certain incident angle, thus the interference of the incident wave with the resonant radiation causes the Fano resonance. As long as the structural shape and parameters of the metasurface are reasonably selected to design an open-loop resonator, it can excite different resonant modes when the incident angle changes, and realize the regulation of transmittance or reflectivity, so as to realize specific mathematical operations [19]. Here, we specifically pay our attention to introduce the Pancharatnam–Berry phase metasurface for optical differential operations.

### 2.1 Pancharatnam–Berry phase

When a vector undergoes parallel transport from one point to the adjacent point, the vector does not rotate relative to the tangent plane formed by the two adjacent points, which means that the vector does not change locally. Consider the parallel transport along a closed path on a three-dimensional (3D) surface, after the vector returns to the starting point, it obtains by a certain rotation. Therefore, this phenomenon of no local change but overall change is actually a geometric phenomenon, and the overall change is related to the geometric path. Polarization and phase are two intrinsic features of electromagnetic waves. Fundamental polarization states, such as linear, circular, and elliptical polarizations, have a spatial homogeneous distribution. In 1892, a prominent geometric representation of polarization known as the Poincare sphere is proposed to describe the polarization state of light as a point on the surface of a unit sphere. When a Stokes vector undergoes a parallel transport along a closed path on the Poincare sphere, after it returns to the starting point, the polarized vector obtains by a certain phase, which is referred as Pancharatnam–Berry (PB) phase [71, 72].

In general, the Stokes sphere is used to describe the evolution of the polarization state in the Stokes parameter space. The parallel transport of the vector on the sphere reveals the inevitable geometric phase of circularly polarized light. The polarization state ψ(R) evolves in the Stokes parameter space, and the geometric phase can be expressed by the corresponding Berry connection and Berry curvature:

(1) $$A^{\sigma }\left(R\right)=-i\psi^{*}\left(R\right)\cdot \left[\nabla_{R}\psi \left(R\right)\right]=-\frac{\sigma }{2\rho }\text{cot }\theta \hat{\varphi },$$

(2) $$F^{\sigma }\left(R\right)=\nabla_{R}\times A^{\sigma }\left(R\right)=\sigma \frac{\rho }{\rho^{3}}.$$

Berry connection and Berry curvature play effective “vector potential” and “magnetic field” respectively in momentum space. In Berry’s theoretical framework, the state undergoes a cycle to move parallel in the parameter space along a closed path, and then returns to the initial state to obtain an additional geometric phase:

(3) $$\Phi_{G}=\underset{C}{\iint }F^{\sigma }\left(R\right)\cdot \text{d}S.$$

In spherical coordinates, the geometric phase can be written as

(4) $$\Phi_{G}=\underset{C}{\iint }\text{sin}\theta \text{ d}k\text{ d}\varphi \text{ d}\theta =-\frac{1}{2}\sigma \mathrm{\Omega .}$$

where σ=+1 and σ=−1 indicate left- and right-handed circular polarizations, respectively. Therefore, the geometric phase can be simply expressed as one-half of the solid angle.

**Figure 3:**
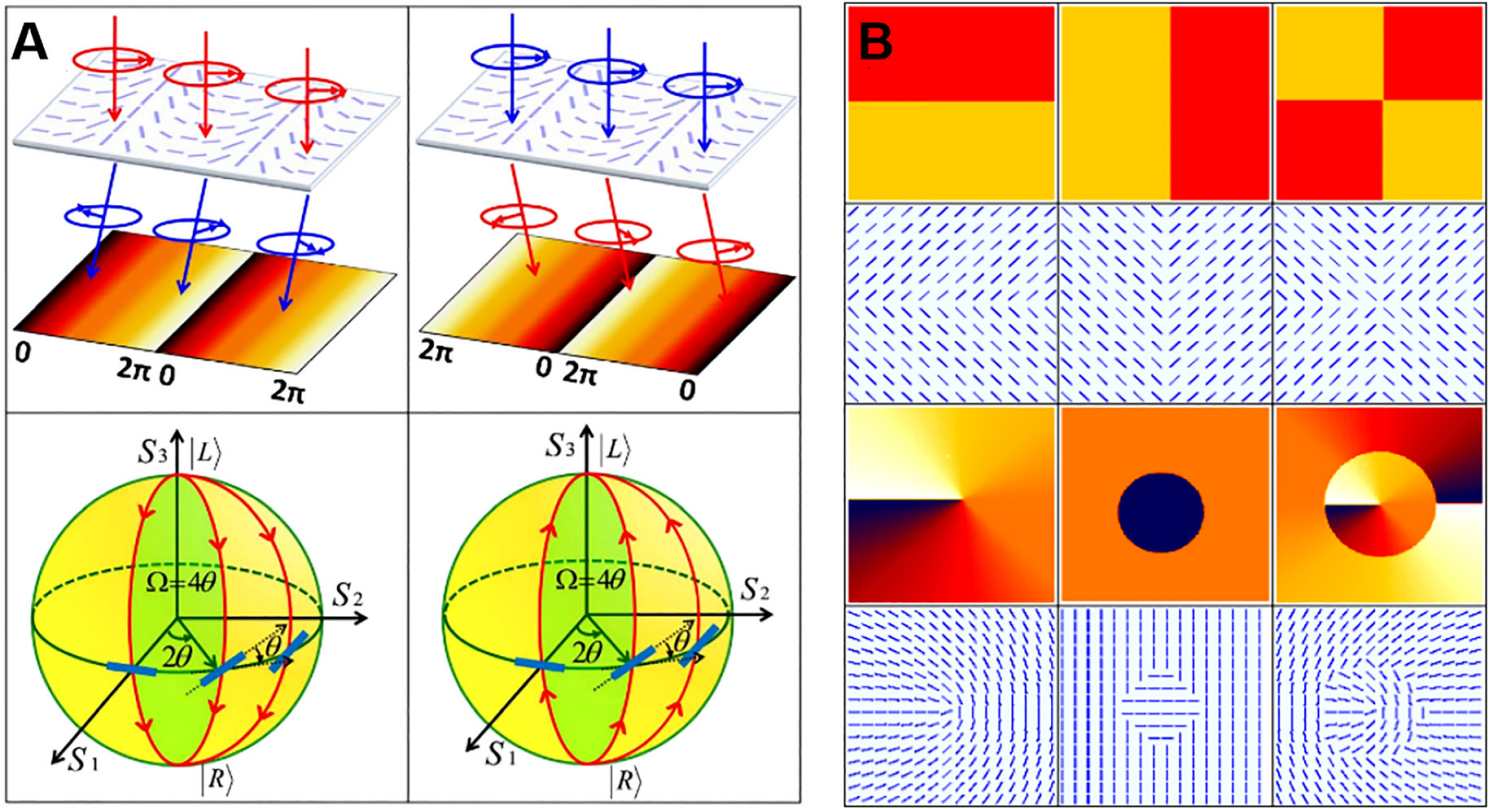
Illustration of the polarization evolutions on Poincaré sphere and the PB phases included [73]. (A) The induced PB phase varies periodically with the rotation of optical axes, and this variation is in the opposite directions when left- or right-circularly polarized light passes through the metasurface. The red and blue arrows indicate left- and right-circularly polarized lights. The incident light gets a 2 θ phase, which is double the orientation angle of the optical axis (slow) and half of the solid angle enclosed by the polarization evolving routes. (B) By controlling the local orientation of the optical axis, any desired phase can be obtained.

Figure 3A shows the relationship between the acquired PB phase and local orientation of the optical axis in the metasurface [73]. The Jones calculus is usually applied to analyze the polarization related optical elements. The Jones matrix of a half-wave plate can be written as [74]

(5) $$J\left(\theta \right)=\left[\begin{array}{ll} \text{cos }2\theta & \text{sin }2\theta \\ \text{sin }2\theta & -\text{cos }2\theta \end{array}\right].$$

where *θ* is the orientation of the optical axis. When a left circularly or right-circularly polarized beam normally impinges onto the metasurface, the output case can be written as

(6) $$E_{\text{out}}=J\left(\theta \right)\cdot E_{\text{L}}=E_{\text{R}}\text{exp}\left(-i2\theta \right),$$

(7) $$E_{\text{out}}=J\left(\theta \right)\cdot E_{\text{R}}=E_{\text{L}}\text{exp}\left(-i2\theta \right),$$

where $E_{\text{L}}=\left(e_{x}+ie_{y}\right)/\sqrt{2}$ and $E_{\text{R}}=\left(e_{x}-ie_{y}\right)/\sqrt{2}$ . We can clearly see that this process brings in an additional spin-dependent phase $\Phi_{G}=-2\sigma \theta$ . Geometric calculations show that the phase is equal to half of solid angle Ω on the Poincaré sphere. Therefore, by controlling the local orientation of the optical axis, any desired phase can be obtained as shown in Figure 3B. The PB phase is only related to the geometric path of the system evolution, and it is independent of the wavelength. Therefore, the PB phase elements have important applications in broadband all-optical image processing.

### 2.2 Dielectric metasurfaces

We focused on the design principle and preparation process of the dielectric metasurface. In order to modulate the polarization state of the beam, a dielectric metasurface composed of continuous or quantized sub-wavelength gratings is designed [49, 75]. The homogeneous grating operates as a uniaxial crystal, with its optical axis parallel and perpendicular to the grating. This means that the electric field along or perpendicular to the grating will obtain a different phase shift. In other words, the light field undergoes so-called shape birefringence through the dielectric metasurface. By designing spatially varying sub-wavelength gratings, polarization can be manipulated at every precise location. A non-uniform metasurface is designed with periodic changes in the direction of the optical axis in one dimension. When the light beam passes through this metasurfaces, PB phase gradient that depends on the spatial rotation rate Ω of the local optical axis will be generated. By appropriately designing Ω , we can obtain metamaterials with positive or negative PB phase gradients. It displays the evolution of PB phase and induced spin-dependent splitting as an element for manipulating the light field [76].

The dielectric metasurface can be manufactured by writing spatially varying nanogrooves in a fused silica sample with a femtosecond laser. The laser beam is focused 200 mm below the surface of the glass sample [77]. Under strong laser irradiation, uniform glass (SiO_2_) is decomposed into porous glass (SiO_2(1−*x*)_ + *x*O_2_), and its refractive index depends on the intensity of the laser [78]. Therefore, periodic changes in intensity can lead to modulation of the refractive index, which can produce grating-like nanostructures and lead to the formation of birefringence in isotropic glass samples. The local optical axis directions (fast axis and slow axis) are respectively perpendicular and parallel to the groove [79]. Since the feature size of the structure is much smaller than the operating wavelength, the manufactured metasurface can be regarded as a birefringent waveplate with homogeneous phase retardation and a locally varying optical axis direction. The phase retardation is $\psi =2\pi \left(n_{e}-n_{o}\right)h/\lambda$ , where h is the writing depth. As a linear approximation, the effective ordinary and extraordinary refractive indices can be written as [79]

(8) $$n_{\text{o}}=\sqrt{fn_{1}^{2}+\left(1-f\right)n_{2}^{2}},\;n_{\text{e}}=\sqrt{\frac{n_{1}^{2}n_{2}^{2}}{fn_{2}^{2}+\left(1-f\right)n_{1}^{2}}}.$$

Here, f is the duty cycle, and $n_{1}$ and $n_{2}$ are the refractive indices of the two media that form the grating-like structure of the metamaterial.

**Figure 4:**
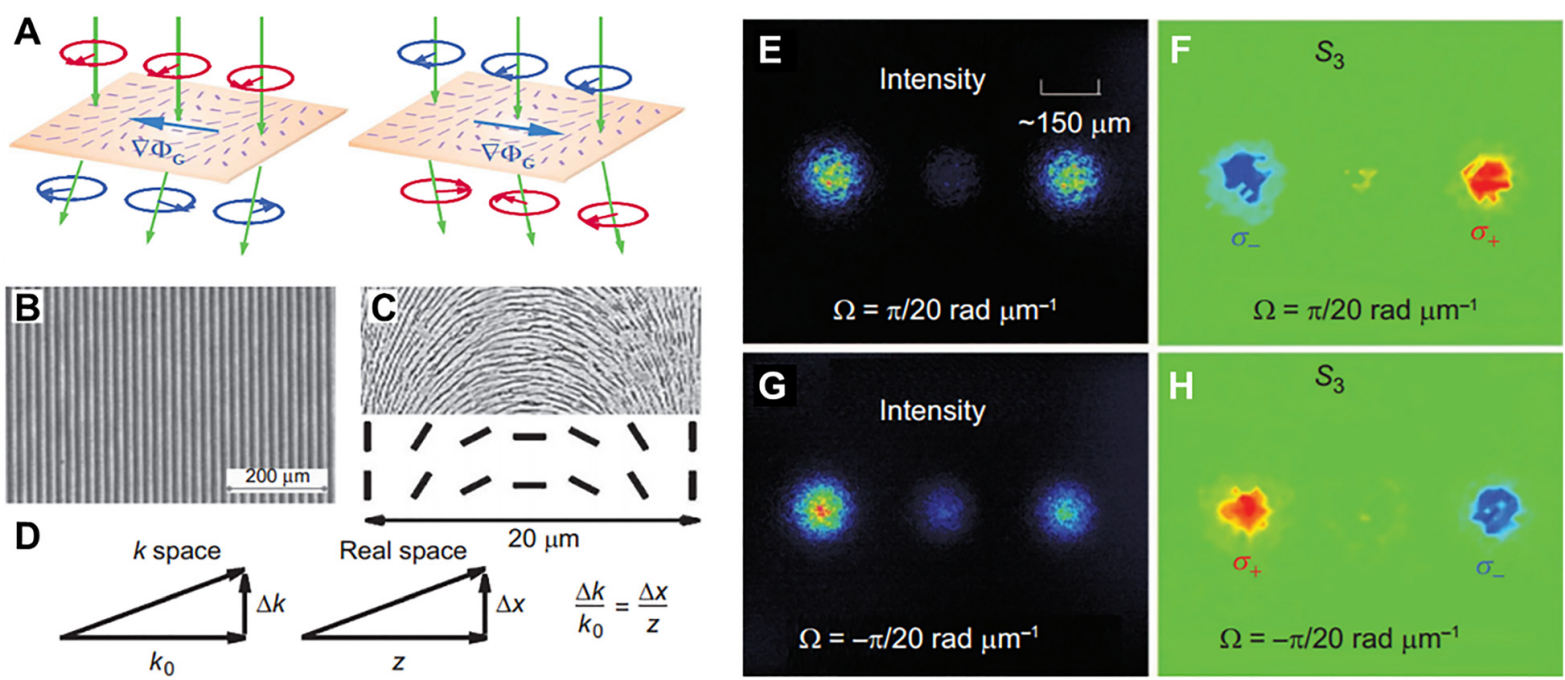
Schematic and experimental demonstration of photonic SHE in a structured metasurface [76]. (A) represent the transition of the spin state when circularly polarized light is transmitted through a structured metasurface with a spin-dependent PB phase gradient $\nabla \Phi_{G}$ . The short line marked on the metasurface indicates the local optical axis direction. The green arrows show the wave vectors. For the incident left-handed circularly polarized light ( $\sigma_{+}$ , red) and right-handed circularly polarized light ( $\sigma_{-}$ , blue), the metasurface produces the opposite $\nabla \Phi_{G}$ and the opposite spin-dependent momentum displacement. (B) Microscopic photograph of the metasurface. (C) A schematic illustration of the detailed geometry and local optical axis (slow axis) of the metamaterial in a period (20 mm). (D) The mapping relationship between momentum displacement Δk and induced real space displacement Δx . After the linearly polarized beam passes through two metasurfaces with completely opposite rotation rates Ω , charge-coupled device records the intensity and the corresponding $S_{3}$ parameters, the observation plane is 10 cm away from the metasurface. The experimental results of the metamaterial with $\Omega =\pi /20\text{ rad μ}{\text{m}}^{-1}$ are shown in (E) and (F). (G) and (H) The experimental results of the metamaterial with $\Omega =-\pi /20\text{ rad μ}{\text{m}}^{-1}$ .

There are two types of geometric phase related to spin–orbit interaction of light: Rytov–Vladimirskii–Berry (RVB) phase and the Pancharatnam–Berry (PB) phase [80], [81], [82], [83], [84], [85], [86], [87], [88], [89], [90], [91], [92], [93], [94]. The RVB phase is related to the change of wave vector direction. The wave vector represents the propagation direction of the beam, so reflection or refraction at the optical interface controls the propagation direction of the beam to change, resulting in different RVB phases. This manifests itself as a spin-dependent spatial displacement of beam centroid in real space (coordinate space). The PB phase is related to manipulating the polarization of light (two circular polarizations with converse chirality), resulting in a spin Hall displacement in momentum space (*k* space). As the beam propagates, the *k*-space displacement causes a real-space displacement. To verify this prediction, a dielectric-based metamaterial was constructed at visible wavelengths (in Figure 4). Relying on the generation of the PB phase gradient in one dimension, the two circular polarizations obtain opposite phases and displacements, thereby observing the photonic spin Hall effect. In the following section, we will discuss the role of photonic spin Hall effect in optical differential operation.

### 2.3 Optical differential operations

Here, we introduce the principle of PB phase gradient metasurface to achieve spatial differentiation. PB phase is related to polarization change and is created by the local orientation of the optical axis of the metasurface [71, 72]. As the orientation of the metasurface is locally variant, the metasurface can be characterized by a position-dependent Jones formalism [95]. The Jones matrix of metasurface with spatially varying local optical axes and constant phase retardance π is J(θ) , as shwon in Eq. (5). If the input electric field is left-handed circularly polarized (LCP). Given the fact that metasurface is located at the Fourier plane, the field distribution is calculated by the Fourier transform of the LCP object light ${\tilde{E}}_{\text{in}}\left(k_{x},k_{y}\right)=ℱ\left[E_{\text{in}}\left(x,y\right)\right]|L$ , where $E_{\text{in}}\left(x,y\right)$ is input electric field in real space, $|L=\left[\begin{array}{l} 1\\ i \end{array}\right]$ . $k_{x}=\frac{x^{\prime }}{\lambda f},k_{y}=\frac{y^{\prime }}{\lambda f}$ are respectively the coordinates in the momentum space, which $x^{\prime }$ and $y^{\prime }$ are the real space coordinates at Fourier plane. λ is the working wavelength and f is the focus length. The transmitted field after the metasurface sample can be determined by

(9) $${\tilde{E}}_{\text{out}}\left(k_{x},k_{y}\right)=J\left(\theta \right){\tilde{E}}_{\text{in}}\left(k_{x},k_{y}\right)={\tilde{E}}_{\text{in}}\left(k_{x},k_{y}\right)\text{exp}\left(i2\theta \right)\left[\begin{array}{l} 1\\ -i \end{array}\right].$$

As we can see, this process brings in an additional phase 2θ , which is the PB phase in nature. Meanwhile, incident LCP turns into output right-handed circularly polarization (RCP). The output field in real space could be written as:

(10) $$E_{\text{out}}\left(x,y\right)=ℱ\left[{\tilde{E}}_{\text{out}}\left(k_{x},k_{y}\right)\right]=E_{\text{in}}\left[\left(x-\Delta \right),y\right]\left[\begin{array}{l} 1\\ -i \end{array}\right].$$

where $\Delta =\frac{\lambda f}{\Lambda }$ represents the beam shift of the output RCP.

Similarly, the input electric field is RCP incident, $|R=\left[\begin{array}{l} 1\\ -i \end{array}\right]$ , the achieved PB phase will be written as −2θ . The sign ‘−’ is due to PB phase spin-dependent property. The handedness of output beam is reversed. And the output after the metasurface can be given as

(11) $$E_{\text{out}}\left(x,y\right)=E_{in}\left[\left(x+\Delta \right),y\right]\left[\begin{array}{l} 1\\ i \end{array}\right].$$

It is well known that a linearly polarized beam can be regarded as the combination of LCP and RCP components. Therefore, for attaining spatial differentiation, we let a linearly polarized beam incident the metasurface, two shifted of LCP and RCP images along the opposite direction are obtained at the image plane, which could be given as:

(12) $$E_{\text{out\_image}}\left(x,y\right)=E_{\text{in}}\left[\left(x-\Delta \right),y\right]\left[\begin{array}{l} 1\\ -i \end{array}\right]+E_{\text{in}}\left[\left(x+\Delta \right),y\right]\left[\begin{array}{l} 1\\ i \end{array}\right].$$

Here, the analyzer is set after the metasurface, which is orthogonal with the input linear polarizers. The output electrical field could be rewritten as:

(13) $$E_{\text{out\_edge}}\left(x,y\right)=\left(E_{\text{in}}\left[\left(x+\Delta \right),y\right]-E_{\text{in}}\left[\left(x-\Delta \right),y\right]\right)\left[\begin{array}{l} 0\\ i \end{array}\right].$$

If the shift Δ is much smaller than the image profile, the output electric field amplitude will be approximately proportional to the first-order spatial differentiation of the input electric field [96]:

(14) $$\left|E_{\text{out\_edge}}\left(x,y\right)\right|≃2\Delta \frac{\text{d}E_{\text{in}}\left(x,y\right)}{\text{d}\left(x\right)}.$$

## 3 All-optical image processing

### 3.1 Pattern recognition and image processing

Pattern recognition has become an important research branch of computer vision technology, which is also an important foundation for image retrieval. Edge is the most basic feature of an image. It contains the most valuable information for human vision and machine vision. Image edge detection greatly reduces the amount of data, and eliminates information that can be considered irrelevant, retaining the important structural attributes of the image. The detection of edge information is an indispensable link before image analysis and recognition. It plays a vital role in image detection and pattern recognition [97], [98], [99], [100]. By using the edge detection algorithm on the target object, the flat area (low frequency information) can be filtered out, and the necessary structural features (high frequency information) can be retained. This greatly reduces the amount of data that needs to be processed for the original image. There are two completely different methods in the field of image recognition: one is digital image edge detection methods [101, 102]; the other is optical image edge detection methods [39, 103, 104].

Digital image processing methods are usually used to realize edge detection with the help of computers. At this stage, the core is to construct an algorithm template for edge detection. For example, convolution filtering is used. Sobel operator is one of the simplest edge detection techniques based on convolution. Sobel operator uses the difference of image pixel brightness to work. But machine language can only recognize numbers, and image information must be converted into digital information for processing. The conversion process requires one-to-one correspondence, which is also complicated and time-consuming. For objects with rich information and inconspicuous intensity characteristics (such as transparent phase objects), the computer processing effect is generally not ideal. However, the optical method is to directly process the optical information. Compared with the digital method, it has the advantages of fast speed, parallel processing and large information capacity. Moreover, the calculation itself is completed by optical passive devices, so the energy consumption of the whole process is low.

### 3.2 Edge-enhanced image

Images are the basis of human vision, and significant changes in image attributes usually reflect important events and changes in image information. These include discontinuities in depth, discontinuities in surface orientation, changes in material properties, and changes in scene lighting. Compared with a flat area, the human eye is more sensitive to edge information, so the edge information of an image occupies a particularly critical position in both human vision and image analysis. The edge is defined as the area boundary where the brightness, phase, polarization, and frequency of the target optical image change sharply. The edge describes the contour of the target object, and the essence of edge detection is to extract the boundary line between the object and the background in the image. The purpose of edge detection is to identify points in the image where the light field distribution changes significantly (discontinuously), which means that the edge detection basically calculates the derivative of this distribution change, that is, the differential operation of the light field. Compared with digital image processing technology, optical edge detection directly calculates multiple parameters of the light field and can selectively extract different material information.

The prototype of optical edge detection technology can be traced back to Zernike’s pioneering work in enhancing the contrast of transparent cells in 1942 [105]. The principle is to approximately transform the phase distribution of the object into the amplitude distribution of the imaging plane, by attenuating the zero-frequency light and shifting the phase with 90°. The proposal of Zernike phase-contrast-enhanced microscopy laid the foundation for modern microscopes, and for this, I won the Nobel Prize in Physics. However, this method has certain limitations. The resulting halo effect seriously affects the authenticity of phase contrast imaging. For this reason, people continue to improve filtering methods to improve image contrast. In 1965, Bracewell et al. proposed a spatial filtering operation based on Hilbert transform and fractional Hilbert transform, which can enhance the edge features of the input object in one direction (one-dimensional (1D)) [106].

In order to achieve isotropic edge enhancement, Jeffrey Davis et al. proposed a theoretical and experimental scheme using radially symmetric Hilbert transform to achieve 2D edge enhancement. The programmable liquid crystal spatial light modulator is loaded with a radial Hilbert phase diagram, that is, a vortex phase structure with topological charge l=1 and spatial phase exp(iφ) , which realizes the 2D edge enhancement of a circular hole. This marks the first proposal of spiral phase contrast (SPC) technology [107]. With the introduction of the SPC technology, researchers have carried out a series of improvements to reduce the edge noise of the image to obtain high-quality edge-enhanced images. For example, a programmable spatial light modulator is used to optimize the phase in the traditional vortex phase plate, and encode into Laguerre–Gauss mode, Bessel-like amplitude modulation mode, Airy beam mode, etc. to improve contrast of edge imaging [108], [109], [110], [111], [112]. SPC technology, as an important edge detection method, plays an important role in enhancing the visibility of amplitude-type and phase-type objects. It is used in microscopic imaging [39, 103, 113], optical interferometry [114], and mapping the phase singularity [115] and other aspects have been widely used.

Except for the spiral phase contrast approach, a series of novel image edge detection approaches such as photonic spin Hall effect [116], [117], [118], Goos–Hänchen effect [119], and the Brewster effect [120, 121] have been proposed based on novel physical mechanisms. Edge detection is achieved using novel optical materials to fabricate filters, including but not limited to metal surface plasmons [122], gratings [70, 123], [124], [125], [126], photonic crystals [127], [128], [129], and metasurfaces [19, 43, 68, 96, 130], [131], [132], [133], [134]. The development of micro–nano processing technology has promoted the development of various new materials and structures, of which the development of metasurfaces is the most prominent. The computing metasurface provides an efficient solution for spatial differential operations due to its compactness, easy integration, and multifunctionality. In the future, computing metasurfaces are expected to gradually replace traditional vortex or amplitude filters in the implementation of optical computing (differentiation and convolution).

**Figure 5:**
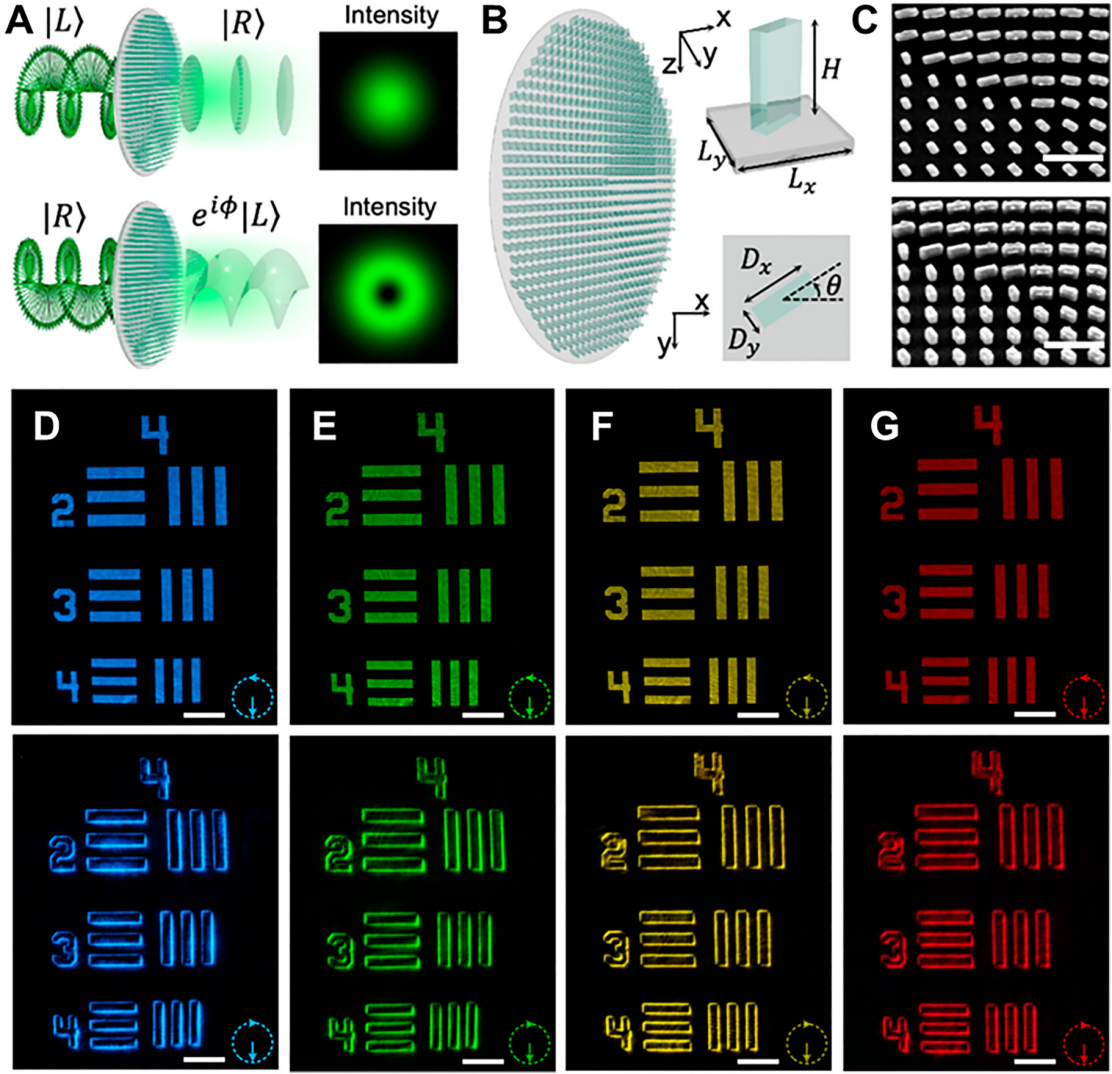
Vortex-phase metasurface for edge detection [132]. (A) The incident circularly polarized light passes through the metasurface, and the polarization chirality is reversed. For the incident RCP, the metasurface will produce a constant phase distribution and intensity distribution on the output beam, realizing a two-dimensional spatial differentiation operation. (B) Schematic of the all-dielectric metasurface spatial filter designed to realize photonic spin-multiplexing function. Inset: Perspective and top view of a metasurface unit cell formed by amorphous TiO_2_ nanopillars on a silica substrate. (C) Top view and oblique view of SEM image of TiO_2_ nanopillar array. Scale bar: 1 μm. (D)–(G) The bright field images of the resolution test chart under the incident LCP light and the edge-enhanced phase contrast images with the incident RCP light at wavelengths of 480, 530, 580, and 630 nm. The inset shows the handedness of the incident light. Scale bar: 100 μm.

A new spin-multiplexed vortex-phase metasurface was designed, as shown in Figure 5. Through special design of different response characteristics to photon spins, this metasurface realized switchable and wide-wavelength edge-enhanced imaging [132]. Titanium dioxide nanoparticles were grown on a silica substrate to form a metasurface. The height of all nanopillars was fixed at 600 nm with a period of 450 nm TiO_2_ was selected as the material for the metasurface because of its high refractive index and low loss at visible light frequencies. The phase shift $\left(\varphi_{x},\varphi_{y}\right)$ is achieved by changing the plane dimensions $D_{x}$ and $D_{y}$ of the titanium dioxide nanopillars, and the geometric phase is controlled by the rotation angle θ relative to the fast axis. Bright-field images are captured under LCP light irradiation, and 2D edge images are obtained in spiral phase contrast imaging under RCP light irradiation. Although using the vortex phase as a filter can achieve the purpose of edge detection, its function is single and cannot perform other basic computing operations such as first-order differentiation. In addition, the filter based on the vortex phase cannot control the resolution of the edge, resulting in limited and uncontrollable detection accuracy.

**Figure 6:**
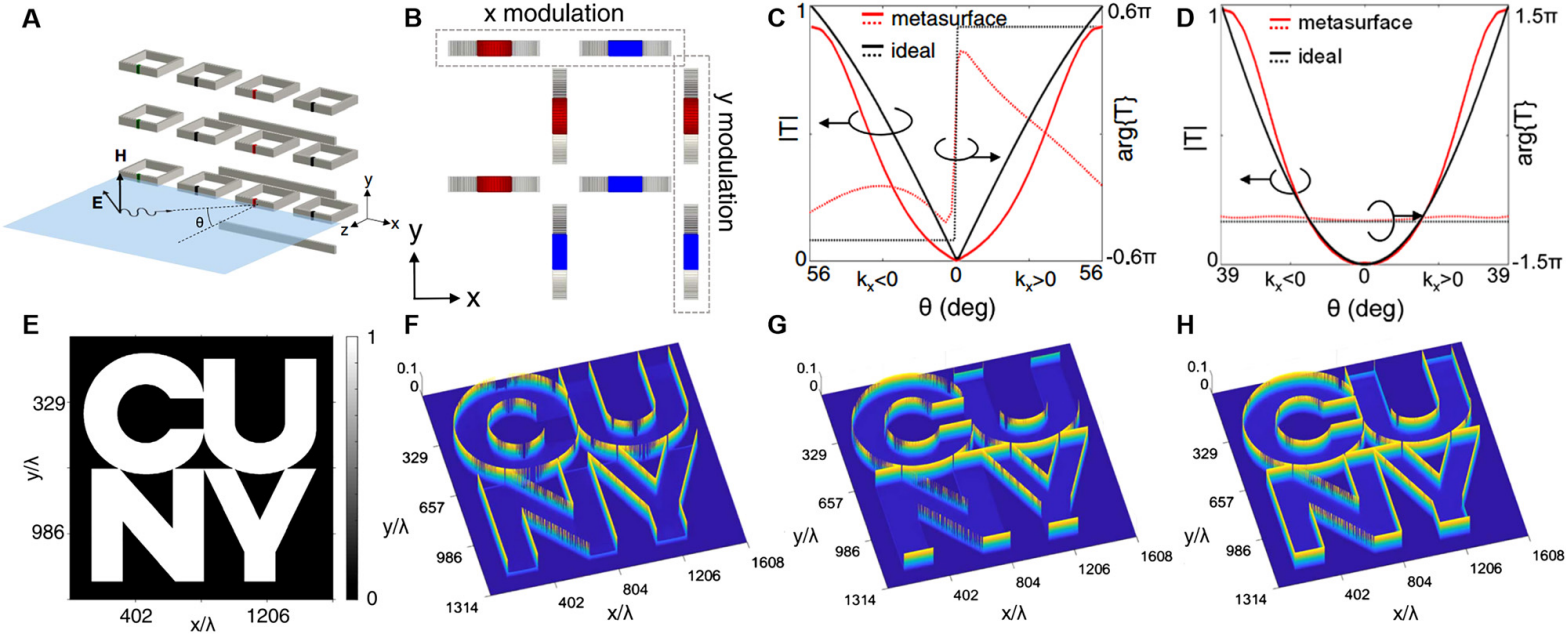
Nonlocal metasurfaces for first-derivative and second-derivative operations [19]. (A) Schematic of a metasurface consisting of a periodic array of resonant particles in the x–y plane, formed by split ring resonators (SRR) parallel to the x–z plane (magnetic dipole moment parallel to the y axis). The metasurface is excited by TM polarized waves propagating in the x–z plane. The metasurface with broken x and z symmetry results in asymmetric responses with respect to $\pm k_{x}$ , achieving the required first-derivative operation. (B) The designed metasurface structure with 90° rotational symmetry to implement the second derivative in 2D. (C) The relationship between the transmission and the incident angle of the metasurface (solid line) and the ideal first-derivative operation (dashed line) in (A). The selected distance of the transmission reference plane results in a 0° transmission phase at normal incidence. (D) The relationship between the transmittance of the metasurface and the incident angle (solid line) for the response to the ideal second-derivative operation (dashed line). The selected distance of the transmission reference plane results in a 180° transmission phase at normal incidence. (E) The input image used to test the response of the metasurface at $f=0.98f_{0}$ , where $f_{0}$ is the resonance frequency of the unmodulated metasurface. In the process of signal processing, the input signal needs to be discretized, and the pixel size in each direction is 0.86λ . (F) The output 1D edge image in the x direction by the metasurface when the x -polarized wave is illuminated. (G) The output 1D edge image in the y direction when the y -polarized wave is illuminated. (H) The 2D edge image obtained when unpolarized light from the normal direction is used to illuminate the metasurface.

The non-locality of metasurfaces can be carefully designed to realize the manipulation of light signals in the momentum domain in ultra-thin spaces to perform basic mathematical operations [19]. In the actual design, slow-period spatial modulation is introduced into the metasurface based on split-ring resonators to improve its nonlocal response amplitude. By properly modulating the parameters, different types of mathematical operations can be realized, such as 1D first-order differential $\left(\frac{\partial }{\partial x}\right)$ , 1D second-order differential $\left(\frac{\partial^{2}}{\partial x^{2}}\right)$ , and 2D Laplace operation $\left(\frac{\partial^{2}}{\partial x^{2}}+\frac{\partial^{2}}{\partial y^{2}}\right)$ . To extend to 2D operation, they designed a metasurface with rotationally symmetrical response. The simplest case is to rotate two identical metasurfaces by 90° and then combine them (as shown in Figure 6). The combined metasurface has 90° rotational symmetry. It is expected to provide the same second derivative response to TM polarized waves on the x and y axes. The ideal Laplace operation has the same effect on TE and TM polarization. If unpolarized light is used to illuminate the target image, the edges of the image in all directions can be well detected. The disadvantage of this method is the use of basic sinusoidal modulation, which imposes restrictions on the complexity of the mathematical operations that can be performed.

We focus on the metasurface design based on the PB phase inserted between two vertically aligned linear polarizers for broadband edge detection [96]. Unlike other spatial differentiation methods, this scheme is based on the spin–orbit interaction. When a linearly polarized plane wave is incident on the PB phase metasurface to produce a phase gradient $\varphi \left(x,y\right)=\frac{\pi x}{\Lambda }$ , Λ represents the period of the metasurface along the x direction, corresponding to the split RCP and LCP components respectively increase in phase +2φ and −2φ . If this metasurface is inserted into the Fourier spectrum surface of an image, the polarization direction of the analyzer is perpendicular to the input linear polarization, the output electric field amplitude is approximately proportional to the first-order spatial differential of the input electric field: $\left|E_{\text{out\_edge}}\left(x,y\right)\right|≃2\Delta \frac{\text{d}E_{\text{in}}\left(x,y\right)}{\text{d}\left(x\right)}$ . In other words, the metasurface splits an input horizontally polarized light beam into LCP and RCP, and there exists a certain lateral displacement of the two beams, which are transmitted along different directions. When the generated displacement $\Delta =\frac{\lambda f}{\Lambda }$ is small enough, the overlapped portion of the LCP and RCP components is still horizontally polarized light. However, the edge part contains only LCP or RCP components, so only the outer edge information can be left after the middle solid part is filtered out by a vertical analyzer. Therefore, we need larger period of the PB phase metasurface Λ for better performance of the differentiator.

**Figure 7:**
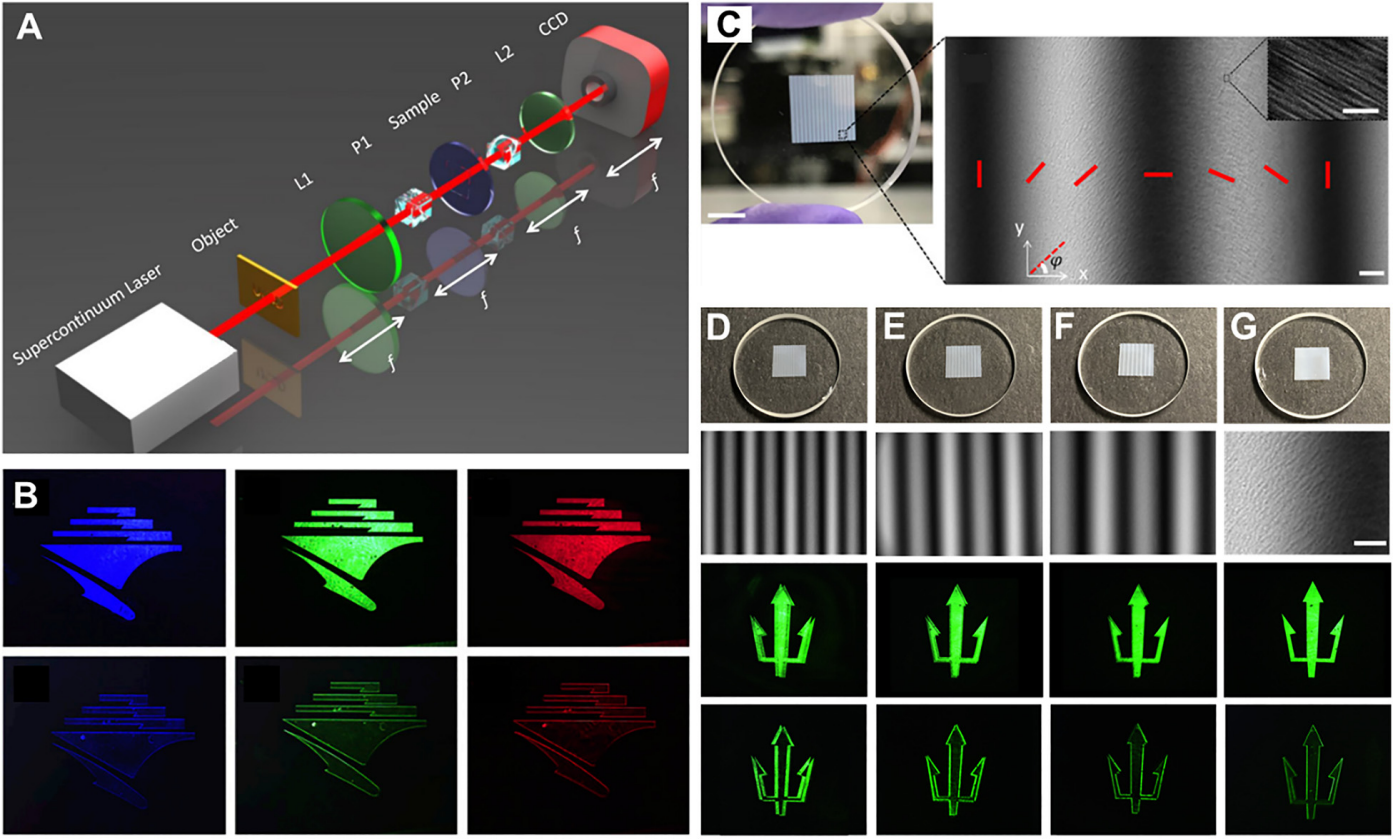
Broadband edge detection based on high-efficiency PB phase metasurface [96]. (A) Experimental setup: two lenses form a 4f system, and the metasurface (sample) is placed between P1 and P2. L: lens, P: polarizer. (B) The metasurface period Λ of the imaging experiment is 8000 μm. The illumination wavelengths are respectively 430, 500 and 670 nm. The first row shows the images without the analyzer after the sample, and the second row shows the edge image with the crossed polarizers. (C) Photograph of a sample with a diameter of 2.54 cm (left), with a metasurface pattern area of 8 × 8 mm (scale bar: 5 mm). The optical image of the marked sample (right), the red lines indicate the direction of the nanostructure in a period. The inset is the SEM image of the nanostructure (scale bar: 500 nm). (D)–(G) Various resolutions of edge detection with different phase gradient periods: 500, 750, 1000, and 8000 μm, at the wavelength of 500 nm. The first row is photographs of different metasurface samples. The second row is polarized images of the samples (scale bar, 125 μm). The third row is two separated LCP and RCP images without the analyzer. The fourth row is edge images corresponding to different resolutions.

In the preparation of metasurfaces, a femtosecond laser is used to process ribbon-shaped nanostructures inside the glass. The diameter of the glass substrate is 2.5 cm, the thickness is 3 mm, and the area of the sample is 8 × 8 mm, as shown in Figure 7C. The metasurface is placed on the Fourier spectrum surface of the 4f system (see Figure. 7A), and the wavelengths are respectively 430, 500, and 670 nm. It not only validates the concept of edge detection, but also proves its broadband performance (see Figure. 7B). The broadband characteristics of the metasurface are derived from the geometric phase of the nanostructure orientation, which is essentially independent of the wavelength. In addition, the detection resolution of the image edge corresponds to different PB phase gradient periods Λ with 500, 750, 1,000, and 8000 μm in Figure 7D–G. The highest resolution of the system is about 2 μm corresponding 8000 μm phase gradient period (Figure 7G), which is close to the diffraction limit of the optical system. The 1D phase gradient metasurface only provides sensitivity to edges along the gradient direction. The metasurface can be extended to 2D edge detection by changing the 1D phase gradient $\varphi \left(x,y\right)=\frac{\pi x}{\Lambda }$ to a radial phase gradient $\varphi \left(x,y\right)=\frac{\pi \sqrt{x^{2}+y^{2}}}{\Lambda }$ . Thanks to the transparent glass element structure and high transmittance, the high-efficiency medium metasurface exhibits obvious advantages over metal metasurface in the transmission mode. Due to the high efficiency of the metasurface, this method plays an important role in image edge detection in some very weak signals.

Except for PB phase metasurface spatial differentiators, liquid crystals can also be used to construct spatial differentiators for tunable edge detection [124, 135]. This method has the advantages of convenient use, simple fabrication process, and real-time controllability. Since the size of liquid crystal molecules is generally in the order of micrometers, the structure of the prepared liquid crystal differentiator is relatively large. However, the metasurfaces based on nanofabrication processes can reach the nanoscale and achieve higher-precision optical field regulation. Performing differential operations on metasurfaces fabricated from ribbon-shaped nanostructures in glass relies on two orthogonal linear polarizers [96]. The differential process for metasurfaces structured as common dielectric pillars depends on the incident circular polarization, and the fabrication of such metasurfaces is more complex and expensive due to electron-beam lithography [132].

### 3.3 Quantum edge detection

**Figure 8:**
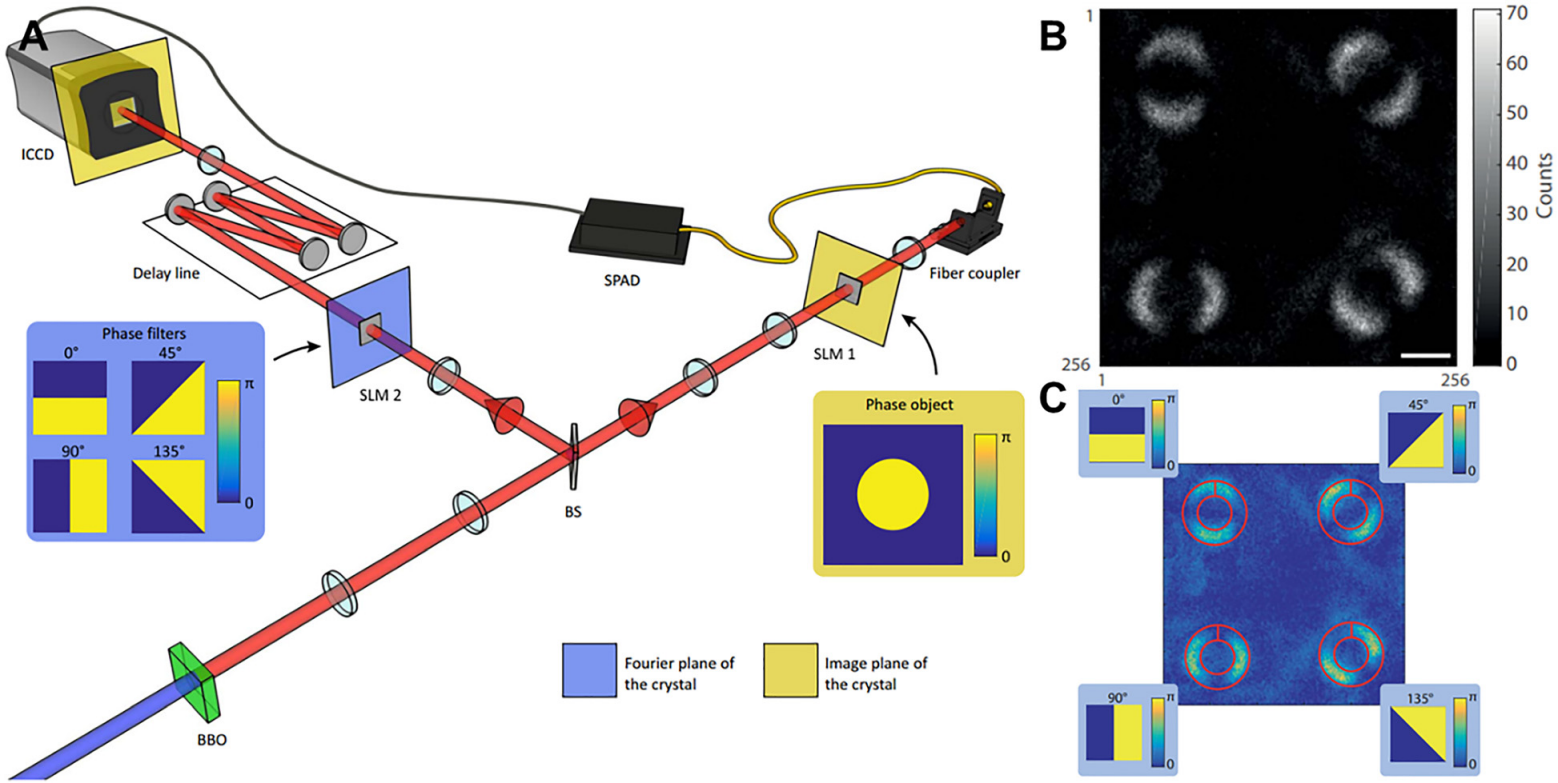
Edge-enhanced image based on quantum imaging violates Bell inequality [42]. (A) Perform imaging settings for Bell inequality test. A β -barium borate (BBO) crystal pumped by an ultraviolet laser serves as a source of entangled photons. The beam splitter (BS) separates pairs of entangled photons. The intensified charge coupled device (ICCD) camera acquires the ghost image of the phase object in spatial light modulator (SLM 1) placed on the first photon path. Four different spatial filters implement nonlocal filtering. These spatial filters and the camera are placed in another optical path. By being triggered by single-photon avalanche diode (SPAD), the camera acquires a coincident image that can be used to perform the Bell test. (B) The coincidence of the same phase circle obtained by using four phase filters in different directions counts a single image. (C) The correspondence between the phase filter used and the specific observation of the object acquired in a single image is highlighted, by a ring-like region of interest (ROI) along the edge of the phase circle object within (B).

Metasurface is a 2D array of ultrathin metallic or dielectric artificial microstructures. It is also a multi-purpose optical element that enables the modulation of the amplitude, phase and polarization of the electromagnetic field to achieve the required electromagnetic field distribution [22, 49, 136, 137]. It provides unique solutions for optical exotic phenomena such as negative refraction, achromatic focusing, and electromagnetic stealth. However, these functions are usually developed for various applications in classical optics. The cross-fertilization of optical edge detection techniques with quantum light sources has led to new and richer phenomena than just the classical field of intense light. In 2009, Padgett et al. at the University of Glasgow exploited the signal generated by the spontaneous parameter down-conversion process of the crystal, the correlation between the position of the idle photon and the orbital angular momentum (OAM). The object and the vortex phase filter are placed in two paths, and then by scanning the coincidence data of the point detectors at the two collection ends, the nonlocal edge-enhanced imaging of the target object was implemented for the first time in the quantum ghost imaging scheme [138]. In 2016, this group demonstrated the first wide-field vortex phase-contrast imaging system with nonlocal properties employing a multipixel array camera instead of a point detector at the imaging end. This proved that the former requires fewer photons to image phased objects than standard phase-contrast imaging techniques [139]. In 2019, they established for the first time that edge-enhanced images can be used to prove that quantum entanglement violates Bell inequality by means of wide-field imaging, building on the original quantum ghost imaging scheme shown in Figure 8 [42].

**Figure 9:**
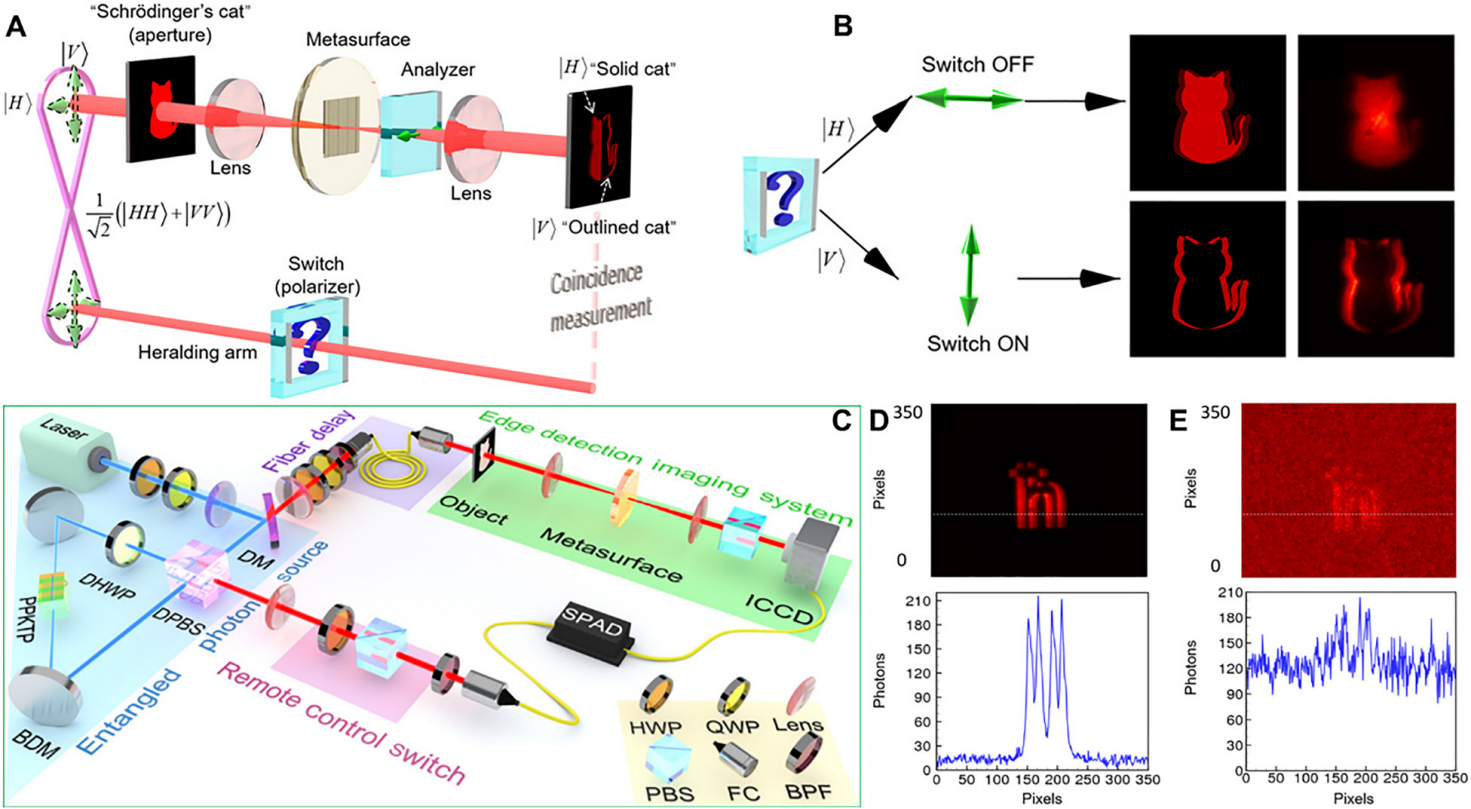
Quantum switchable edge detection based on PB phase metasurface [43]. (A) Schematic of experimental setup for switchable quantum edge imaging. (B) The polarization switch controls the coincidence image: Polarization |H> is OFF state, corresponding to the bright field mode “solid cat” (the first row shows simulation and experimental imaging); |V> state is ON state, corresponding to edge detection mode “outlined cat” (the second row shows simulation and experimental imaging). (C) Experimental setup for metasurface enabled quantum edge detection. BDM, broadband dielectric mirror; PPKTP crystal, periodically polarized KTiOPO4 crystal; DPBS, dual-wavelength polarization beam splitter; PBS, polarization beam splitter; DM, dichroic mirror; FC, fiber coupler; BPF, band-pass filter; DHWP, dual-wavelength 1/2 wave plate; QWP, 1/4 wave plate; SPAD, single photon avalanche detector; ICCD, intensified charge coupled device. The blue (red) light path presents the 405 nm (810 nm) light. The edge detection switch is on the forecast end, and the edge detection system is installed on the imaging end. (D) Coincident edge image of photon pair in external trigger mode and the one-dimensional intensity distribution along the white dashed line. (E) Edge image in internal trigger mode and the one-dimensional intensity distribution along the white dashed line.

We have reviewed the implementation of 1D optical spatial differentiation based on PB phase gradient. Edge detection of images can be achieved by inserting the metasurface between two orthogonal polarizers. Here, we present the application of 1D computing metasurface in quantum entanglement sources. Recently, the quantum edge detection [43] has been proposed and demonstrated by a combination of a high-quality polarization entanglement source based on the Sagnac ring structure [140, 141] and a highly efficient dielectric metasurface [96]. The imaging mode was remotely switched between normal mode and edge detection mode by changing the polarization state of the trigger photon and the coincidence measurement of image. The higher signal-to-noise ratio of entangled photon illumination compared to direct single-photon illumination was also demonstrated under weak field illumination. In the case of classical light field illumination, this artificial computing metasurface realizes first-order partial differentiation of electric field and 1D edge detection. The Schrödinger’s cat pattern is used as an input target object, as shown in Figure 9A. The analyzer is oriented in the same direction as the polarizer, the output is a “solid cat”. The analyzer is orthogonal to the polarizer, the final output is an edge image “contour cat” whose absolute value of the electric field can be approximated as a first-order spatial differentiation of the input object. Next, the case of introducing a polarization-entangled light source is discussed. If a polarization-entangled source containing photons of unknown state is used for illumination, and the deflector is removed, the image will be a mixture of quantum states, constituting the “Schrödinger’s cat” state, i.e., the “outlined cat” and the “solid cat” states.

The concept of “Schrödinger’s cat” is used to illustrate the expected performance of switchable quantum edge detection. As shown in Figure 9B, the edge detection imaging system is independent of the entanglement source, the precursor and the coincidence measurement component. When the incident photon is horizontally polarized (green arrow), the illuminated target object crosses the metasurface and is then split into LCP and RCP images by a pre-designed horizontal shift. The overlapping LCP and RCP components will pass through the horizontal polarization detector, resulting in a complete “solid cat”. If the incident photons are vertically polarized (green arrow), the overlapping LCP and RCP components will be recombined into linearly polarized components and completely blocked, leaving only the edges, resulting in a “silhouette cat”. In this study, a quantum switchable edge detection is achieved by using a quantum state of 1/2 ( |HH>+|VV> ) polarization entanglement as the photon source. The coincident image between the entangled photon pairs is obtained by introducing a predictor as an external trigger. When the imaging and prediction photons are entangled and their polarization states are not known, the image states (“solid cat” and “outlined cat”) before the measurement are also in an unknown mixed state. If the polarization state of the incident photons is triggered and post-selected by the photons measured by the projection at the prediction end, the final image switches between the regular mode of the “solid cat” and the edge detection mode of the “outlined cat” to the determined one.

The schematic diagram of the experimental setup is shown in Figure 9C. The polarization entangled photons are generated by the spontaneous parametric down-conversion process in a type II phase-matched periodically polarized KTiOPO4 (PPKTP) crystal. Compared to other ways of generating photon pairs, such as atomic systems and solid-state quantum dots, crystal-based photon sources are simpler in structure, require fewer components, and have higher brightness and signal-to-noise ratios. The pump light comes from a continuous single-frequency diode laser at 405 nm (Toptica, TOP mode-405-HP_40116). To obtain a better spatial pattern, the pump light is first coupled into a single-mode fiber. The laser from the fiber coupling mirror is first passed through a 1/2 waveplate (HWP), a 1/4 waveplate (QWP) and a polarization beam splitter (PBS) to obtain a pure horizontally linear polarization. A combination of the QWP and HWP is then used to balance the pumping power and relative phase in both clockwise and counterclockwise directions. The pump light is focused by a pair of optimized focal length lenses to obtain a beam waist of about 40 μm at the center of the crystal. A PPKTP crystal with a length of 20 mm is embedded in the Sagnac interferometer. The temperature of the PPKTP crystal is set to 17 °C, the temperature at which the photon simplification wavelength is matched, by means of a home-made temperature controller with a stability of 0.002 °C. Two broadband dielectric mirrors and a dual-wavelength (405 and 810 nm) PBS form the self-stabilized Sagnac interferometer. The dual wavelength 1/2 waveplate placed in the Sagnac ring is fixed at a 45° angle to keep the pump light horizontally polarized in front of the crystal in the counterclockwise cycling direction, while meeting the Type II quasi-phase matching condition. The down-converted photon pairs pumped by the two bidirectional recirculating beams are separated by a dual-wavelength PBS and collimated by two lenses. One of the photons enters the imaging system through the fiber coupler, and the other enters the forecast side, i.e., the remote switch control side.

In the current experimental system, when the forecast photon signal is used as an external trigger source, the temporally uniformly distributed noise can only accumulate within a short compliance time window, ensuring that only a very small number of noisy photons fall into the effective compliance. In classical optics, however, the noise accumulates over time. In Figure 9D, the edge image is acquired by an SPAD-triggered ICCD camera with a very low dark count. In contrast, for the internal trigger mode, the camera exposure is continuous and the total exposure time is set to the same as the external trigger time. In addition, other experimental conditions were consistent for image acquisition in both modes of operation, including a continuous-wave light source, low photon flux, and signal light of comparable intensity to the background noise. In the direct imaging scheme, both signal and background photons are detected indiscriminately, so the signal-to-noise ratio of the images is low, as shown in Figure 9E. We also intercepted the 1D intensity distribution at the white dashed position of the image for quantitative analysis. Our experimental scheme for quantum edge detection improves the signal-to-noise ratio by at least one order of magnitude compared to direct imaging. Alternatively, if a pulsed laser source is synchronized with the camera, a similar signal-to-noise ratio can be achieved, but it lacks nonlocality.

It is experimentally demonstrated that the polarization entanglement photon source can be used as a non-local switch for the optical edge detection mode without any manipulation of the 4f imaging system based on an efficient dielectric super-surface. By projecting the idle photons on the prediction end onto the |H⟩ or |V⟩ polarization state, we can selectively trigger the ICCD to acquire a normal image or an edge image, respectively, which can be considered as a quantum edge detection switch. This quantum edge detection scheme also provides a new solution for secure image communication such as encryption and steganography, which cannot be achieved using conventional light sources. On the other hand, our remote switching edge detection scheme using predictive single-photon imaging provides a high signal-to-noise ratio. Although not exceeding the scattering noise limit, it still shows some advantages for photon number-constrained imaging and sensing applications.

## 4 Microscopy imaging applications

### 4.1 Differential interference contrast microscope

Biological samples are usually transparent, and dyes will affect the activity of cells. In order to enhance the optical imaging process, by placing appropriate optical elements in the incident light path, the input light field can be changed with phase, amplitude, and spatial frequency on the sampling plane [3]. Loading a vortex phase on the Fourier plane can realize the edge extraction of the phase object. Early research mostly used spatial light modulators to realize the vortex phase [39, 103, 108, 142], [143], [144], [145], [146]. In recent years, metasurfaces with micro–nano structures have also been used to achieve edge extraction based on vortex phase [132, 134]. Based on this operation, many methods to improve phase contrast have been extensively studied, such as phase contrast imaging and differential interference contrast (DIC) imaging [147], [148], [149], [150], [151], [152].

The physical principle of DIC microscope is completely different from phase contrast microscope. The structure of the DIC microscope includes four special optical components: polarizer, DIC prism, DIC glider and analyzer. The polarizer is installed directly in front of the condensing system to produce linearly polarized light. In the condenser, a quartz Wollaston prism, or DIC prism, is installed. It can split a beam of light into two beams with different polarization directions ( x and y directions), which form a small angle. Initially, the two beams are in the same phase. After passing through the adjacent area of the specimen, the thickness and refractive index of the specimen are different, causing the optical path difference of the two beams. A second Wollaston prism, a DIC glider, is installed at the back focal plane of the objective lens. It combines the two light waves into one beam. At this time, the polarization planes ( x and y polarization) of the two beams still exist.

Before the beam forms the DIC image of the eyepiece, the direction of the analyzer and the polarizer are orthogonal. Finally, the beam passes through the analyzer and interferes. The optical path difference between the x and y polarized waves determine the light transmittance of the analyzer, which can change the brightness of the image. When the optical path difference is 0 , the light cannot pass through the analyzer; when the optical path difference is equal to half the wavelength, the light passing through the analyzer reaches the maximum value. In order to achieve the best image contrast, the optical path difference can be changed by the vertical fine adjustment of the DIC glider. Therefore, on the gray background, the structure of the specimen shows a difference in lightness and darkness. It is usually bright on one side and dark on the other side, which gives a colorless and transparent specimen a relief-like three-dimensional impression. DIC microscope makes the observation effect of cell structure obvious, especially some larger organelles, such as cell nucleus and mitochondria. At present, micromanipulations such as gene injection, nuclear transfer, genetic modification, etc. are often performed under this microscope.

**Figure 10:**
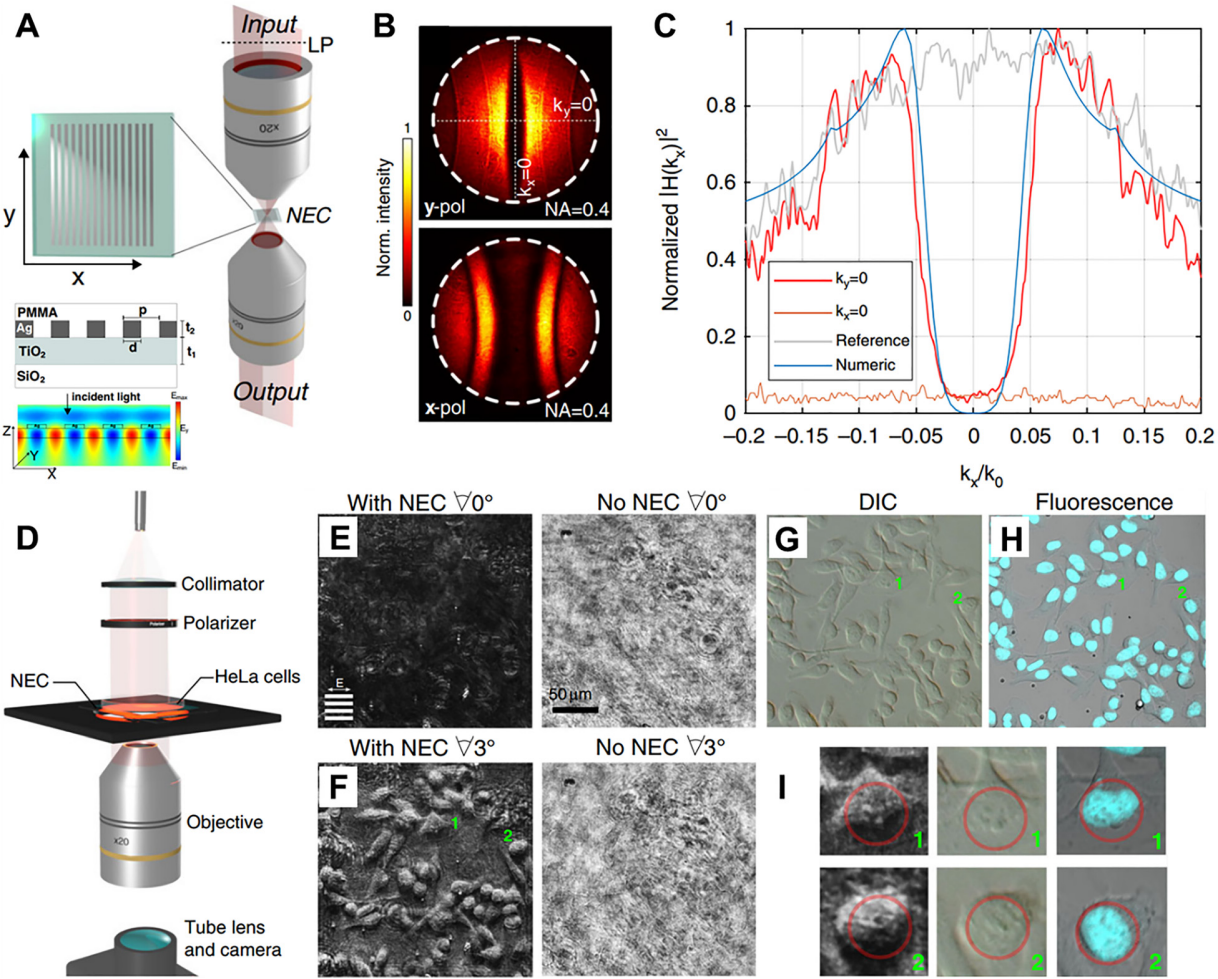
Nanophotonics enhanced coverslip (NEC) for high-contrast intensity images of phase objects [126]. (A) Measuring equipment for optical transfer function of fabricated NEC. The linear polarizer selects the desired polarization direction relative to direction of the grating fringes on the NEC (inset marked by dotted line). The output lens produces a Fourier plane image. The other inset reveals the nanostructure of NEC cross-section, in which design parameters are $t_{1}=100$ nm, $t_{2}=40$ m, d=200 nm, and p=400 nm. When the light of wavelength λ=650 nm is incident vertically, the simulated electric field distribution ( $\hat{y}$ component) of the induced standing wave in the waveguide is displayed. (B) The normalized two-dimensional modulation transfer function measured when the incident light (wavelength of λ=637 nm) is polarized parallel to ( y -pol) and perpendicular to the silver grating fringe ( x -pol). (C) The cross-sectional view along the dotted line (red) in (B), which highlights the suppression of low k in the direct beam. The corresponding simulated profile of the s -polarization (blue) and the spatial frequency of the incident beam (grey) are given for comparison with experimental measurements. (D) Experimental device for phase object microscopy imaging: The wavelength of the light source is 637 nm, and the incident light field whose polarization direction is parallel to the grating line is generated through the collimator and polarizer. The culture dish of Human cancer cells (HeLa) is fixed on top of NEC. (E)–(F) Images obtained with the same cell area with and without NEC under two different tilts (no tilt in (E), tilt 3° in (F)) relative to the incident light. (G)–(H) A conventional microscope image of the same cell using DIC and fluorescence. The blue area in the fluorescence image highlights the cell nucleus. (I) The magnified image shown by the number in (F) to (H) for NEC tilted at 3° , DIC and fluorescence microscope. The circle highlights the nucleolus in each cell nucleus.

Compared with traditional systems, the use of nanophotonic differentiators can significantly reduce the size, opening new doors for optical analog image processing in computer vision applications. As shown in Figure 10A and B, the 2D spatial differentiator that directly distinguishes the edges in the image is composed of Si nanorod photonic crystals [129], which can convert the image $E_{\text{in}}$ to its second derivative $E_{\text{out}}/\nabla^{2}E_{\text{in}}$ , where $\nabla^{2}=\frac{\partial^{2}}{\partial x^{2}}+\frac{\partial^{2}}{\partial y^{2}}$ is a Laplacian operator. The use of 2D photonic crystals allows differentiation and edge detection in all directions, with numerical aperture (NA) up to 0.315 and experimental resolution less than 4 μm. By switching to the working wavelength, the high-contrast cell boundaries are clear observed. The nanophotonic differentiator is directly integrated into the optical microscope and camera sensor, proving that it can be easily integrated vertically into existing imaging systems.

Next, the imaging system is further reduced by using the matelens as the focusing element. The integration of the differentiator and the metalens realizes the entire processing system as a single-chip composite planar optical device. In this case, there is more noise in the image compared to experiments using a differentiator on the objective lens or camera sensor. The additional noise maybe attributed to the diffraction artifacts of the metalens caused by the non-uniform focusing efficiency. This problem is slightly exaggerated due to the weak signal strength after passing through the differentiator. This problem can be minimized by optimizing lens design and manufacturing [153], [154], [155], [156]. Composite nanophotonic systems show the advantages of thinness and the ability to realize complex transfer functions, and can open up new opportunities in applications such as biological imaging and computer vision. This differentiator does not depend on polarization, and a specific wavelength is required to perform the differentiation operation. Therefore, the realization of broadband edge detection is still flawed.

The ability to visualize transparent objects (such as living cells) is at the core of understanding biological processes. The phase contrast microscope can observe weakly absorbing microorganisms without staining or fixing. A new type of nanostructured thin film device has been developed that may replace the bulky optical devices used in traditional phase contrast microscopes. A nanophotonics enhanced coverslip (see Figure 10) has been proposed, which can generate high-contrast images of pure phase objects during transmission [126]. The nanophotonic devices use the contrast forming characteristics of the spatial frequency filter to reject the non-scattered wave components in the sample, but transmit these components with a non-zero phase gradient. Sensitivity to the direction of propagation is achieved by buried waveguides that sample and interfere with each wavefront over a large distance across the surface. This compact device is capable of real-time and all-optical generation of pseudo three-dimensional images of phase objects during transmission. By placing unstained human cancer cells on the device, the internal structure of the cells can be clearly seen. This research demonstrates the great potential of nanophotonic devices for integration into compact imaging and medical diagnostic equipment.

### 4.2 Computing metasurfaces enabled DIC microscope

**Figure 11:**
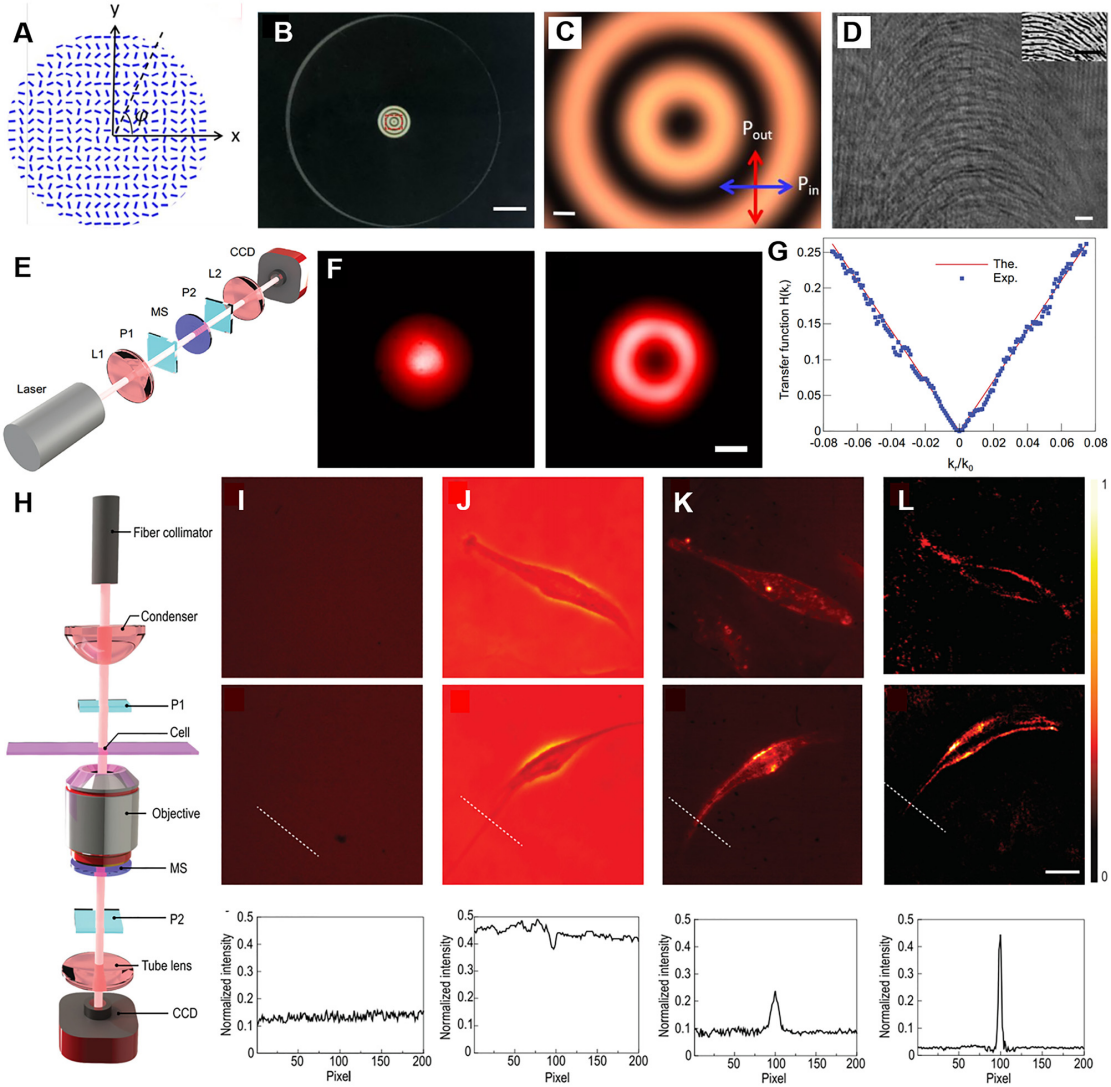
Two-dimensional optical spatial differentiation and high-contrast imaging based on PB phase metasurface [133]. (A) Schematic of the optical slow axis distribution of the metasurface designed to realize the differential operation. (B) Polariscope image of the metasurface. Scale bar, 4 mm. (C) An enlarged image of the sample pattern area marked in (B), under cross-polarized light. Scale bar, 200 μm. (D) shows the finer structure of the metasurface. White scale bar, 3 μm. Inset is top view of SEM. Black scale bar, 1 µm. (E) Experiment setup for measurement of the spatial transfer function: L, lens, focal length 25 mm; P1 and P2, a pair of crossed polarizers; MS, metasurface, period 1000 μm; CCD, charge couple device. (F) The experimental result without and with the spatial differentiator, respectively. Scale bar, 500 μm. (G) The theoretical and experimental 1D transfer functions along the radial direction. (H) Measurement setup for edge detection of a phase object. (I)–(L) Observation methods for transparent cells include bright field (I), phase difference (J), dark field (K), edge detection (L). The first row, the examined human umbilical vein endothelial cells (HUVEC). The second row, the observed human bronchial epithelial cells (HBEC). Scale bar, 100 μm. The third row is the horizontal intensity distribution along the white dashed line.

A new design metasurface owns a symmetric phase gradient along the radial direction, which enables the linearly polarized beam splitting to LCP and RCP components along the radial direction and guarantees the 2D spatial differentiation [133]. The diameter of metasurface sample with patterned area is 4 mm in the center of a 1-inch SiO_2_ substrate, as shown in Figure 11B. The thickness of the substrate is 3 mm. The metasurface pattern was fabricated by scanning a femtosecond pulse laser inside the silica slabs (50 μm beneath the surface). More sample fabrication details could be found in previous works [96]. The polariscope image of the sample (see Figure 11C and D) reflects the form-birefringent characteristics of the metasurface area. The measured constant value of phase retardance of the metasurface sample is π , which works as a half wave plate and ensures the conversion efficiency of the incident linearly polarized beam to RCP and LCP components [95]. The dielectric metasurface based on geometric phase without any resonance structure ensures the operation at broadband working wavelength (whole visible range), enabling differentiation of color images. For our metasurface, the measured conversion efficiency is close to unity at the working wavelength.

For the current 2D edge-detection, it can be regarded as superposition of infinite 1D edge detection processes that radially span the entire 2π azimuth angles in polar coordinate. As shown in Figure 11A, the simulated orientation of the optical slow axis follows the relation of $\varphi \left(x,y\right)=\frac{\pi \sqrt{x^{2}+y^{2}}}{\Lambda }$ ranged from 0 to π , where Λ=1000 μm is the period of metasurface. when the metasurface sandwiched between two orthogonal polarizers is placed on the Fourier plane of a 4f system (Figure 11E–G), the shift △ is much smaller than the image feature size, the output electrical field for the 2D case under polar coordinates can be given as

(15) $$E_{\text{out\_edge}}\left(r,\theta \right)≃2\Delta \frac{\text{d}E_{\text{in}}}{\text{d}r}.$$

Here, $\Delta =\frac{\lambda f}{\Lambda }$ . λ is the working wavelength, f is the focal distance. For an intensity object $E_{\text{in}}\left(r,\theta \right)=A\left(r,\theta \right)$ , A(r,θ) is the amplitude of the input electric field. While for a phase object, $E_{\text{in}}\left(r,\theta \right)=\text{exp}\left[i\Phi \left(r,\theta \right)\right]$ . The final electric field can be further expressed as

(16) $$E_{\text{out\_edge}}\left(r,\theta \right)≃2\Delta \frac{\text{d}\Phi \left(r,\theta \right)}{\text{d}r}.$$

For transparent and colorless objects, the phase information is more useful than the information contained in the amplitude. However, conventional imaging systems are inconvenient to observe transparent objects, especially biological tissues such as cells. The stain affects the viability of cells. The challenge of observing dynamic biological processes is to achieve clear imaging of clear cells. The 2D edge detection metasurface is inserted into the traditional microscope system so that high-contrast images of transparent objects can be realized, as shown in Figure 11H–L. Different imaging techniques were compared with human umbilical vein endothelial cells and human brain endothelial cells grown in tissue culture vessels in order to evaluate the effect of edge detection on the proposed metasurface. Compared with dark field and phase contrast technology, the method of computing metasurface combined with the microscope showed clear and strong signals at the edge of the cell, indicating that the detection of transparent biological specimens has extremely high sensitivity and precision. However, this 4f system requires a series of optical components, and its integration needs to be improved.

**Figure 12:**
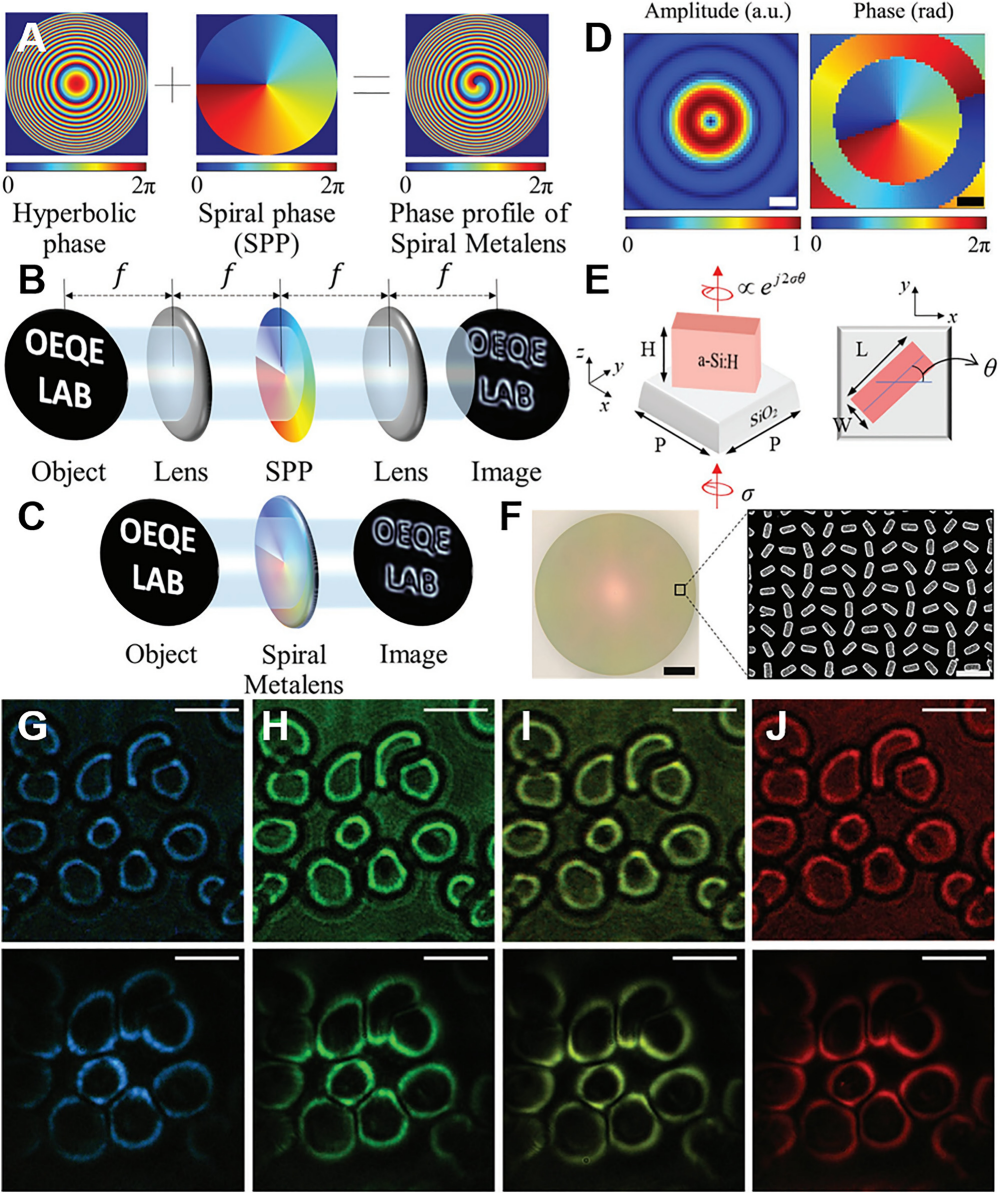
Spiral metalens for edge-enhanced imaging [134]. (A) The phase profile of a single-layer spiral metalens is the sum of the hyperbolic phase and the spiral phase with a topological charge of 1. (B) Simplified optical device for traditional spiral phase contrast imaging. The spiral phase plate (SPP) is placed on the Fourier plane of the 4f system to produce an edge-enhanced image. (C) The spiral metalens is integrated by the lens and the spiral phase plate, which has a higher resolution to show the edge enhancement effect. (D) Numerically calculated amplitude and phase distribution of the spiral metalens on the Fourier plane. Scale bars, 250 nm. (E) The cell structure of the metalens is composed of a-Si:H on a SiO_2_ substrate. The height, length, and width are *H*, *L*, and *W* respectively. The pixel period of the unit cell is *P*. *θ* is the orientation angle of a-Si:H nanorods. When circularly polarized light ( σ , right circular polarization +1, left circular polarization −1) is incident on the unit structure, the cross-polarized component of the transmitted light undergoes a phase change of 2σθ . (F) Image of the manufactured spiral metalens with the diameter of 1 mm. Scale bar, 200 µm. The inset is the SEM image of the top view. Scale bar, 500 nm. (G)–(J) Bright-field images and edge-enhanced images of red blood cells using a ×50 objective lens at wavelengths of 497, 532, 580, and 633 nm. Scale bar, 10 µm.

A compact single-layer spiral matelens based on PB phase is proposed to perform two-dimensional isotropic edge-enhanced imaging [134]. As shown in Figure 12A and F, the phase profile of matelens is the sum of the hyperbolic phase and the spiral phase (spiral phase plate, SPP) with a topological charge of 1 as follows: $\varphi \left(r,\theta \right)=\frac{2\pi }{\lambda }\left(f-\sqrt{r^{2}+f^{2}}\right)+\theta$ . Here φ is the total phase profle, (r,θ) are the polar coordinates at the lens plane, *λ* is a design wavelength, and f is a focal length, respectively. Unlike the traditional 4f Fourier filter system, this solution compresses the imaging and edge enhancement functions into a single-layer metalens (see Figure 12B and C). A metalens sample with a diameter of 1 mm was made using hydrogenated silicon (a-Si:H) placed on a silicon dioxide (SiO_2_) substrate. The spiral metalens with the target wavelength of 580 nm is manufactured by electron beam lithography. The numerical aperture (NA) is determined to be 0.8 at the target wavelength to provide submicron resolution. Utilizing high NA characteristics, the spiral metalens achieves high-resolution imaging up to 0.78 µm submicron level in the broadband visible spectrum, combined with a compact size. Relying on compactness and high-resolution characteristics, this solution is considered to provide a stepping stone for biomedical imaging technology and analog computing. Since the geometric phase method works when the incident light is circularly polarized, some problems will arise with polarization-sensitive samples. Experiments show that the polarization conversion efficiency of vertically incident circularly polarized light has an average value of about 52% . Polarization-insensitive metasurfaces with multi-wavelength or broadband achromatic properties may be a solution. However, it is challenging to achieve both broadband and high NA characteristics at the same time.

We analyze current approaches to reduce the complexity of optical microscopy systems from the perspective of integration processes. The differentiators involving new mechanisms are proposed, such as not relying on two crossed polarizers, which will effectively reduce the number of optical components [126, 129, 157]. Imaging lenses are integrated with differentiators to further reduce the complexity of optical imaging systems [31, 129, 134]. In addition, our future work is to insert two crossed polarizers directly into the microscope system to enable two-dimensional edge detection, even in combination with a quantum entanglement source, to realize a new type of high-contrast quantum microscope.

### 4.3 Quantum differential microscope

Quantum microscope is an important tool to characterize the microstructure and understand the dynamics of living system with quantum illumination [158, 159]. The sensitivity and resolution of the conventionally used microscopes are fundamentally limited by environmental noise, which can be effectively reduced by increasing the intensity of the illumination light. For photo-sensitive biological samples, however, the biophysical damage typically arises due to the large illumination intensities [160, 161]. Recently, the importance of improving sensitivity has attached considerable efforts to apply quantum entanglement photons in optical microscopy [162, 163]. For some biological specimens, the thickness and refractive index inhomogeneity determine how much light scattering it produces. This class of specimens is referred to as phase objects, as they affect significantly only the phase of the input field and not the amplitude. Too little scattering from the specimen makes it challenging to reveal the structure from an overwhelming input light background [164, 165].

We have shown that the operation of first-order differentiation based on geometric phase metasurfaces. The high-brightness polarization entangled light source based on the Sagnac structure uses the nonlocal characteristics to demonstrate a 1D edge detection switch for optical images that can be nonlocally controlled. In the experiment, the polarization state of the photon at the forecast end was controlled remotely, and the image was collected by coincidence measurement at the imaging end. The edge mode and the normal mode of the image can be selectively extracted remotely without any operation on the imaging end. In addition, compared to the traditional direct detection method of internal triggering, the coincidence measurement method that we use correlated photons for external triggering has a higher signal-to-noise ratio. This high signal-to-noise ratio is beneficial to the biological field, such as tracking enzyme reactions, observing biological tissues or photosensitive cells, and other scenes that require extremely low light.

The quantum differential microscope can be developed by incorporating the computing metasurface, quantum-correlated source, and conventional bright-field microscope. The differential image of biological samples can be obtained in low light environments. Compared with coherent light sources and illumination light sources, quantum entangled light sources have less damage to biological cells and their activities. The computing metasurface combined with the traditional optical microscopy has achieved 2D high-contrast images of transparent cells. The quantum edge detection solution provides a new idea for the subsequent development of a new integrated quantum optical image processing system, and also offers a new way for image security communication such as image encryption and information hiding. In addition, the organic combination of metamaterials and quantum optics has also enriched the intersection of different disciplines and created more new opportunities in biological applications.

## 5 Conclusions and perspectives

We have reviewed the physical mechanism of the mathematical differential operation, the design and preparation process of the computing metasurface based on the micro–nano processing technology. It further briefly describes the specific method of realizing all-optical image edge detection by using computing metasurface. The dimensions of detection in a wide working frequency band include both 1D edge detection and 2D edge detection. In real-time image processing applications, such as medical surgery, smart cars, etc., digital high-throughput edge detection is extremely time-consuming. In response to this problem, the use of computing metasurfaces for image edge detection has become an important alternative. Compared with traditional Fourier optical devices, computing metasurfaces benefit from the characteristics of high speed, low power consumption, and monolithic integration. All-optical edge detection devices based on computing metasurfaces are more compact, with higher control accuracy, and have the potential to be integrated into image sensing systems, such as portable digital cameras or biological microscope systems [157, 166].

Since real objects are often 3D and have certain depth information, how to realize edge detection of complex 3D objects is still a difficult challenge [167]. In addition, as the complexity of object information and scenes increases, higher requirements are placed on the fineness and contrast of edge information. Moreover, the edge detection for long-distance objects has potential applications in remote sensing imaging, astronomical observation and other scenarios. However, there is currently a lack of reports in this area, and it is still one of the urgent issues to be solved.

Further miniaturization and integration will be the future development direction of spatial optical analog computing devices. Tunable or multifunctional computing metasurfaces will also further promote the development of this field towards practical applications. In recent years, deep learning and neural networks have promoted the vigorous development of technologies such as image recognition, machine translation, and autonomous driving. Computing metasurfaces can be used as the front or back end of neural networks to further broaden the application of artificial intelligence. With the aid of the fast information transmission speed of the newly developed and deployed 5G network, the spatial analog optical computing is helpful to the development of big data, cloud computing, and the Internet of Things. In the future, it is believed that with the further development of micro–nano processing technology and meta-material design methods, the accuracy and speed of computing metasurfaces will be further improved. In some areas, it can replace traditional electronic computing devices and get more extensive applications.
